# Short-term repeated HRV-16 exposure results in an attenuated immune response *in vivo* in humans

**DOI:** 10.1371/journal.pone.0191937

**Published:** 2018-02-15

**Authors:** Rebecca M. Koch, Matthijs Kox, Corné van den Kieboom, Gerben Ferwerda, Jelle Gerretsen, Sandra ten Bruggencate, Johannes G. van der Hoeven, Marien I. de Jonge, Peter Pickkers

**Affiliations:** 1 Radboudumc, HB, Radboud Institute for Molecular Life Sciences, Department of Intensive Care Medicine, HB, Nijmegen, The Netherlands; 2 Radboud center for Infectious Diseases (RCI), HB, Nijmegen, The Netherlands; 3 Radboudumc, HB, Radboud Institute for Molecular Life Sciences, Department of Pediatrics, HB, Nijmegen, The Netherlands; 4 NIZO food research BV, ZB, Ede, The Netherlands; Public Library of Science, UNITED KINGDOM

## Abstract

**Introduction:**

Naturally, development of adaptive immunity following HRV infection affects the immune response. However, it is currently unclear whether or not HRV re-exposure within a short time frame leads to an altered innate immune response. The “experimental cold model” is used to investigate the pathogenesis of HRV infection and allows us to investigate the effects of repeated exposure on both local and systemic innate immunity.

**Methods:**

40 healthy male and female (1:1) subjects were nasally inoculated with HRV-16 or placebo. One week later, all subjects received HRV-16. Baseline seronegative subjects (n = 18) were included for further analysis.

**Results:**

Infection rate was 82%. Primary HRV infection induced a marked increase in viral load and IP-10 levels in nasal wash, while a similar trend was observed for IL-6 and IL-10. Apart from an increase in IP-10 plasma levels, HRV infection did not induce systemic immune effects nor lower respiratory tract inflammation. With similar viral load present during the second HRV challenge, IP-10 and IL-6 in nasal wash showed no increase, but gradually declined, with a similar trend for IL-10.

**Conclusion:**

Upon a second HRV challenge one week after the first, a less pronounced response for several innate immune parameters is observed. This could be the result of immunological tolerance and possibly increases vulnerability towards secondary infections.

## Introduction

In the recent decade, it has become clear that bacterial sepsis may induce an immunosuppressed state called “sepsis-induced immunoparalysis”[1, 2], a form of immunological tolerance which renders the host unable to clear primary infections leading to increased vulnerability towards secondary infections[3]. This immunologically tolerant state is characterized by both innate as well as adaptive immunodysfunction, such as functional defects in leukocytes, downregulation of cytokines and immunostimulatory membrane-bound receptors, accompanied by the upregulation of negative costimulatory molecules[3, 4]. A similar phenomenon is observed with virulent respiratory virus infections, such as influenza, which predispose to secondary bacterial or fungal infections[5, 6].

Human rhinoviruses (HRVs) are the most frequent cause of the common cold[7, 8] with prevalence estimates as high as 80% of the adult population[9]. HRV infection results in the production of inflammatory cytokines and chemokines[10–14], and subsequent recruitment of immune cells into the nasopharyngeal[15] and bronchial mucosa, and secretions[11–14]. In addition, HRV-specific neutralizing antibodies are produced, resulting in seroconversion approximately 28 days later[11, 16]. HRVs consist of three species, HRV-A, HRV-B and the more recently discovered HRV-C[7]. Due to its high virulence, HRV-C can cause systemic and severe respiratory infections in previously healthy subjects[7]. This has mainly been reported for children, although several adult cases have also been described[7, 17]. Although HRV-A and HRV-B may cause severe infections in young children, immunocompromised patients, and/or patients with pre-existing respiratory diseases[18–21], it is unknown to what extent these species induce systemic immunological effects or lower respiratory tract disease in healthy adult subjects. Furthermore, although *in vitro* studies indicate that HRV can also induce immunosuppressive or tolerance mechanisms[22, 23], this has never been investigated in humans *in vivo*.

In the present study, we investigated the local respiratory tract and systemic immune response following challenge with HRV-16 (a HRV-A species) using the so-called “experimental cold model”. This model is widely used to investigate the pathogenesis of HRV infection, including the effects on pulmonary function[24], allergies[10], chronic obstructive pulmonary disease (COPD)[25], and asthma exacerbations[24, 26]. Subjects were re-challenged with the same virus one week after the first challenge, to investigate whether the primary HRV infection modulates the innate immune response against the second HRV exposure. A one-week interval was chosen, as at this time point the innate immune response induced by the first infection is expected to be largely resolved and specific antibodies have not yet been produced to a large extent[16, 27, 28]. We recently demonstrated that seropositivity for HRV is associated with a virtually nullified immune response upon HRV challenge [29]. Therefore, we only analyzed data from seronegative subjects in the current study.

Our data indicate that, despite similar viral loads upon both challenges, a second HRV challenge one week after the first results in less pronounced response of several innate immune parameters. This could be the result of immunological tolerance and may be associated with increased vulnerability towards secondary infections.

## Materials and methods

### Subjects

This randomized, placebo-controlled study was part of a larger trial also investigating effects of serostatus and gender on the HRV-induced immune response[29]. This study is registered at ClinicalTrials.gov (NCT01823640, participant recruitment and follow-up: March-May 2013). The authors confirm that all ongoing and related trials for this intervention are registered. As this was a pilot study, no power calculation was performed. After approval by the local medical ethics committee CMO Arnhem-Nijmegen (NL42503.091.12; CMO 2012/476), 40 healthy, non-smoking, male and female subjects (ratio 1:1), aged 18–35 years gave written informed consent to participate in the study. All study procedures were in accordance with the declaration of Helsinki. Subjects were screened and excluded if they had a (febrile) illness within four weeks before the HRV-challenge, a pre-existent lung disease, or a history of allergic rhinitis. Subjects were not allowed to take (prescription) drugs throughout the study.

### Study design

**Fig 1:**
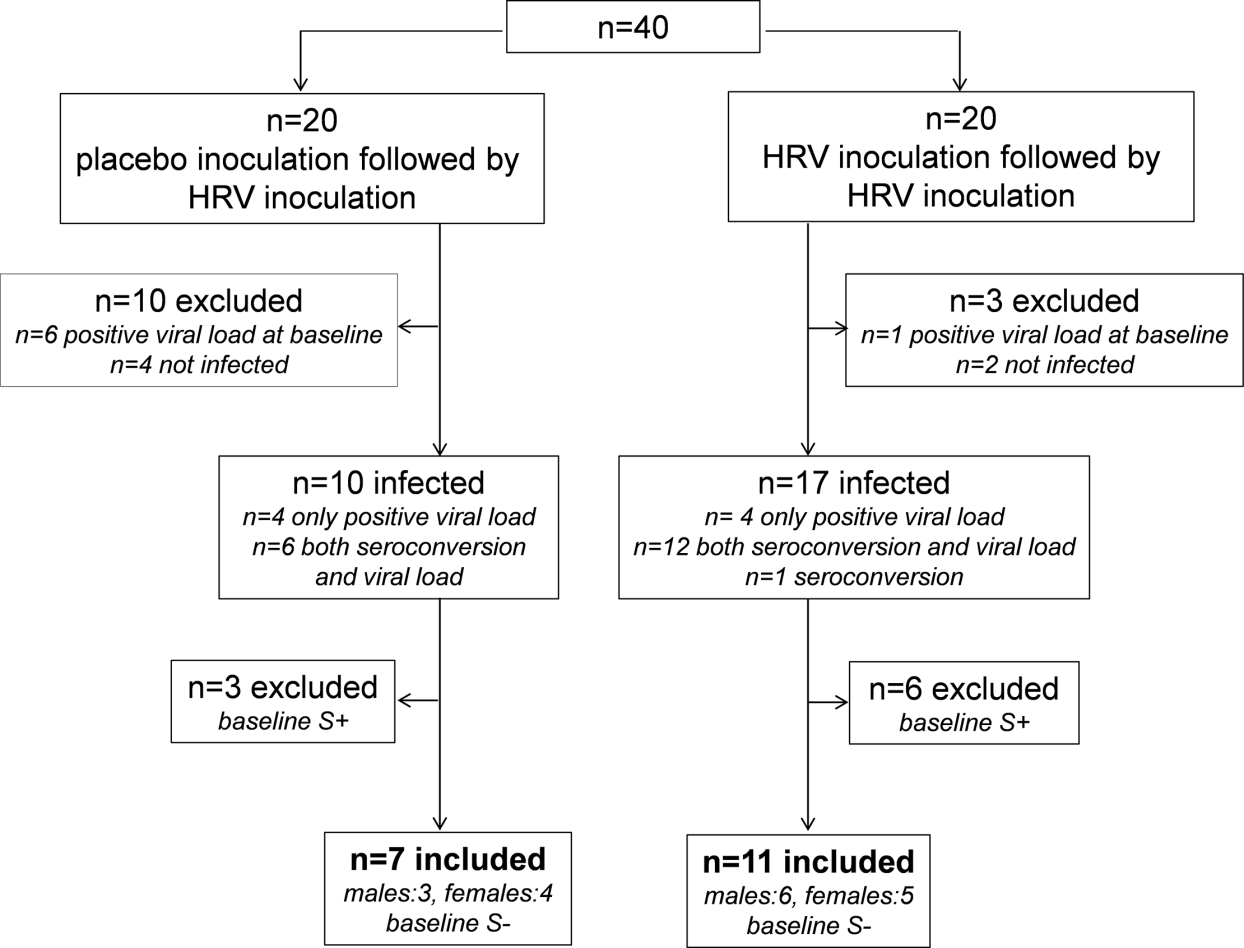
Flowchart of the study. S+ indicates seropositive at baseline; S- indicates seronegative at baseline.

**Fig 2:**
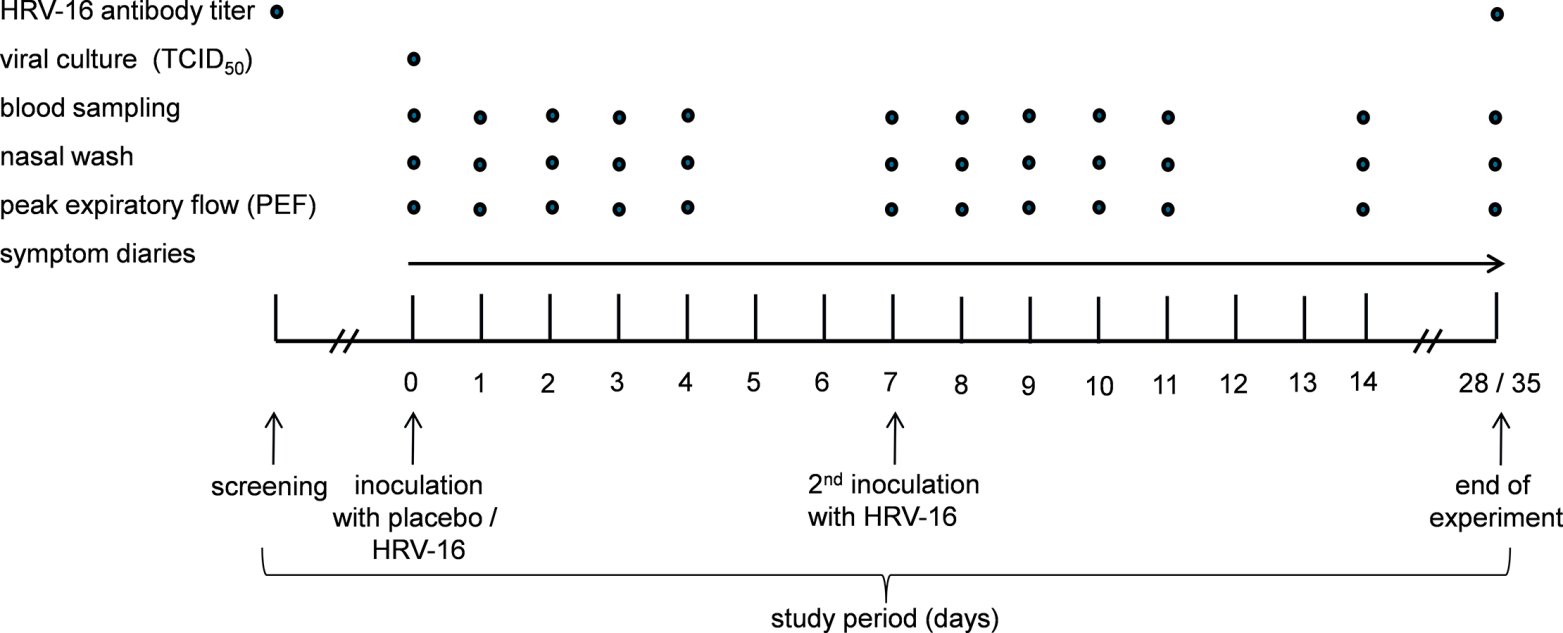
Experimental design of the study.

A flowchart of the study, based on infection rate (further detailed in ‘antibody titer analysis’ section) is depicted in Fig 1 and the study design is depicted in Fig 2. Stratification was based on sex and serostatus as follows: Subjects with the same sex were grouped in two groups of 10 subjects (2x n = 10 males; 2x n = 10 females; in total n = 40) subjects. Each group of 10 subjects consisted of roughly 50% seronegatives and 50% seropositives, based on the baseline antibody titers to HRV-16, measured at the screening visit (assay and cutoff value detailed in “antibody titer analysis” section below). Subsequently, one group of the same sex (n = 10) was randomized to the placebo-HRV group, while the other (n = 10) was randomized to the HRV-HRV group. Randomization was carried out by an independent nurse using the sealed envelope method. Subjects were inoculated by an independent nurse with either HRV-16 by instillation of 10^2^ TCID_50_ (Tissue Culture Infectious Dose in 50% of subjects, based on previous studies[30]) units of HRV-16, diluted in 0.5 mL 0.9% saline, (n = 20; 10 males; 10 females), or placebo (0.9% saline; n = 20; 10 males; 10 females) into each nostril. Subjects remained in the recumbent position for two minutes after instillation and refrained from touching their nose for 30 minutes. Seven days later, all 40 subjects underwent challenge with HRV-16. For the analyses described in this manuscript, only seronegative subjects were included, as serostatus was shown to exert profound effects on the HRV-induced immune response, which was virtually absent in seropositive subjects[29]. Furthermore, we did not stratify our analyses for gender, as we found no differences in any parameters between males and females[29].

### HRV-16 virus

Safety-tested Good Manufacturing Practice (GMP+) grade inoculum pools of HRV-16 were supplied by Respivert Ltd. (MM#400472 Lot#R2011038, London, UK). Each cryovial contained 0.2 mL of HRV-16 at a dose of 2*10^2^ TCID_50_ units/mL and was diluted in Hartmann’s solution to a concentration of 10^2^ TCID_50_ units/mL and stored at -80°C. Per infection day, one aliquot of HRV-16 was cultured in fourfold on a MRC-5 cell monolayer in 10-fold dilutions of samples, positive and negative controls in order to assess infectivity. Cell plates were placed at 33°C with 5% CO_2_ for seven days and observed for the development of cytopathic effects (CPE). The Reed-Muench formula was used to calculate the viral titer[31]. Viral titers were all in the expected infectivity range (10^1^−10^2^ TCID_50_ units/mL) and positive and negative control wells positive and negative for CPE, respectively.

### Antibody titer analysis

A standard end-point neutralization assay for HRV-16 was used to quantify levels of HRV-neutralizing antibodies in the serum of every subject at baseline (virus neutralization titer [VNT] <1:4) and seropositive subjects (VNT ≥1:4) to exclude subjects who were seropositive to HRV-16 for the analyzes described in this manuscript, to assess seroconversion (≥fourfold increase in antibody titer at day 28 post-challenge; day 28 in the HRV-HRV group and day 35 in the placebo-HRV group; Fig 1). The assay was performed on HEL cells at 37°C and 5% CO_2_. Two-step serum dilutions starting at 1:4 were incubated with 10^2^ TCID_50_ HRV-16 for 1 h at 37°C before inoculation on HEL cells. Serum controls were included on each plate to test for toxicity, and a positive control of anti-HRV-16 antiserum was added to each test plate. The formation of CPE was examined daily and after 7 days the cultures were scored for CPE. The Reed-Muench formula was used to calculate the antibody titer[31]. Cells with >50% CPE/well were scored antibody negative and ≤50% CPE as antibody positive. HRV-infection was defined as positive viral load and/or seroconversion (≥fourfold increase in antibody titer at day 28 post-inoculation) compared with baseline[24].

### Nasal wash

Nasal washings for viral load and cytokine quantification were collected daily from subjects according to the method described by Naclerio[32]. Nasal wash from two nostrils was pooled, centrifuged (6000 rpm, 4°C, 20 minutes), and stored at -80°C until analysis.

### Viral load

Non-specific HRV viral load was determined from nasal wash as described previously[33] and performed at the laboratory of medical microbiology at the Radboudumc according to QCMD regulations. Briefly, nucleic acids were extracted from each sample using the MagNA Pure LC (with Total Nucleic Acid Isolation Kit) and PCRs were performed on the LightCycler 480 with Probes Master Mix (Roche Diagnostics, Almere, The Netherlands) using commercial validated primer and probe-mixes (Diagenode, Liège, Belgium; Table B in S1 File). Cycling conditions were 95°C for five minutes, followed by 40 cycles of 95°C (15 sec), 55°C (15 sec) and 72°C (20 sec). Virus amount was recorded semi-quantitatively based on the cycle threshold value (Ct value). All samples in which virus was detected (Ct<40) were considered as having a positive viral load. For samples in which no virus was detected, the Ct value was set as 40 to allow fold change calculations. Fold change from baseline (Ct = 40) was calculated using the formula 2^∆Ct^.

### Symptoms

We previously showed that the HRV-16 batch used in this study does not result in significant induction of cold symptoms[29]. Therefore, we focused on lower respiratory tract and systemic symptoms in the current study. To assess these, all subjects filled out an online symptom diary (LimeSurvey Project Hamburg, Germany), using questions from the validated Wisconsin Upper Respiratory Symptom Survey-24 (WURSS-24)[34], indicative for a lower respiratory tract/systemic respiratory infection (headache, sinusitis, cough, chest tightness, dyspnoea, fever, shivering, body ache and malaise). Symptoms were assessed prior to viral/placebo challenge and then twice daily, to take into account circadian variations, until day 28. The severity of each symptom was rated on a six-point scale.

### Peak Expiratory Flow

To evaluate whether HRV influenced pulmonary function (lower respiratory tract effects), Peak Expiratory Flow (PEF) was measured during each visit using a peak flow meter (PFM20, Omron Healthcare Europe B.V., Hoofddorp, The Netherlands). PEF was determined twice during each visit and the highest value was used. An affected lower respiratory tract was defined as a >20% decrease in PEF of the predicted values of their corresponding age, gender, and stature[35].

### Cytokine analysis

Nasal wash and ethylenediaminetetraacetic acid (EDTA) anticoagulated blood samples were collected at various time points (Fig 1) centrifuged (6000 rpm, 4°C, 20 minutes) and stored at -80°C until analysis. Concentrations of interferon (IFN)-γ, tumor necrosis factor (TNF)-α, interleukin (IL)-1β, IL-6, IL-8, IL-10 were measured using a simultaneous Luminex assay (Milliplex; Millipore, Billerica, MA, USA). Interferon gamma-induced protein (IP-10) was measured using ELISA (R&D Systems, Minneapolis, MN, USA). Lower detection limits were 3.2 pg/mL for IFN-γ, TNF-α, IL-1β, IL-6, IL-8, IL-10, and 156 pg/mL for IP-10.

### Statistical analysis

According to the Kolmogov-Smirnov test, all data were non-normally distributed. Therefore, demographic data are presented as medians [interquartile range] and between-group comparisons were made using Mann-Whitney U tests. All other data are presented as geometric mean and 95% CI. Because there was an interindividual variation in the peak of acute infection ranging from day 1–4 after HRV inoculation, differences were analyzed on log-transformed peak levels in the first four days post-challenge using paired and unpaired Student’s t-tests. Statistical analyses were performed using Graphpad Prism version 5.0 (Graphpad Software, San Diego, CA, USA). Two-sided p-values <0.05 were considered statistically significant.

## Results

### Baseline subject characteristics

Baseline characteristics of the entire study population (n = 40, before exclusion of subject as detailed below) are listed in Table A in S1 File). There were no significant differences in baseline characteristics between the groups. No adverse events occurred during the trial.

### Infection rate

A flowchart based on infection rate is depicted in Fig 1. Seven subjects displayed a positive viral load in nasal wash before challenge with HRV, hence they were excluded from all subsequent analyses. In 27 of the remaining 33 subjects (82%), HRV challenge resulted in infection (positive viral load and/or seroconversion; antibody titers of all subject provided in Table C in S1 File). The six subjects who showed neither positive viral load at any of the time-points post-challenge, nor seroconversion were categorized ‘not infected’ and were also excluded from all subsequent analyses. Furthermore, as explained in the introduction and materials and methods section, 9 subjects who were HRV-seropositive at baseline were excluded from the subsequent analyses. Characteristics of the 18 remaining baseline HRV-seronegative infected subjects who were included for the final analysis showed no significant differences between groups (Table 1).

**Table 1:** Demographic characteristics of the 18 seronegative subjects who displayed positive infection that were included in the final analysis. Parameters were assessed during the screening visit. M: male, F: female. BMI: body mass index. Data are presented as medians [interquartile range].

	placebo-HRV n = 7 M (n = 3) F (n = 4)	HRV- HRV n = 11 M (n = 6) F (n = 5)	total group n = 18	p value between groups
Age (yrs)	**22[21–26]**	**22[22–25]**	**22[21–25]**	**0.51**
Height (cm)	**175[166–182]**	**175[168–184]**	**175[168–183]**	**0.82**
Weight (kg)	**73[66–88]**	**71[64–78]**	**72[66–79]**	**0.56**
BMI (kg/m2)	**23[22–25]**	**23[21–24]**	**23[22–25]**	**0.41**

### Viral load

**Fig 3:**
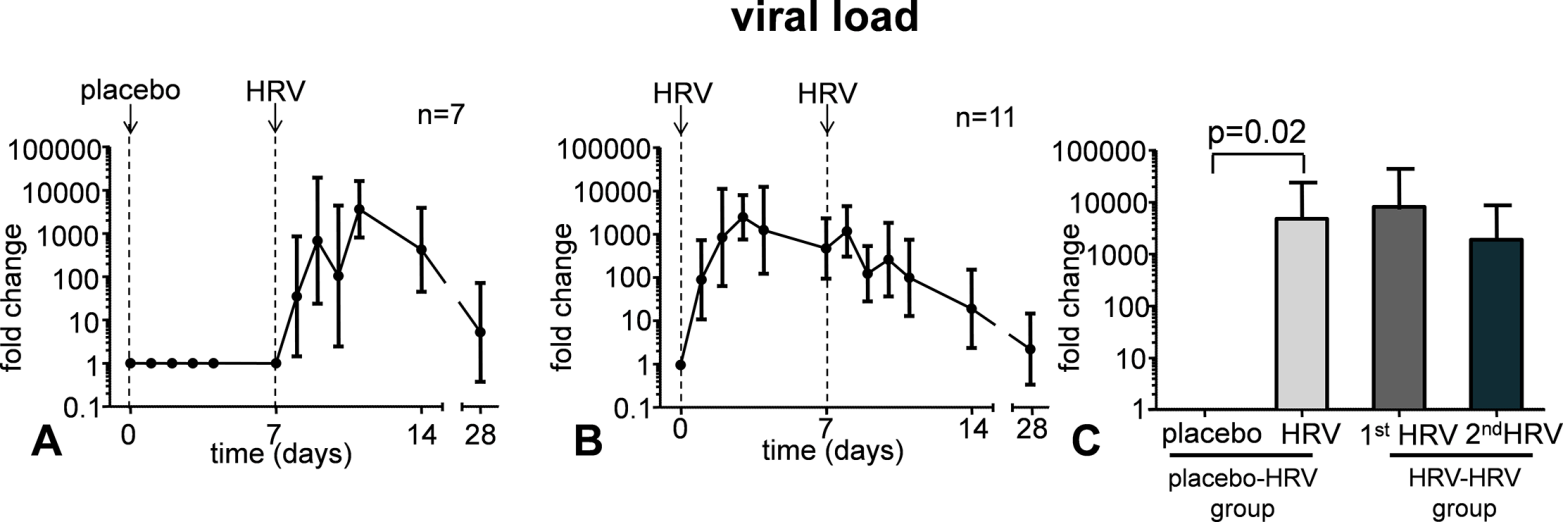
Viral load levels in nasal wash following placebo inoculation and HRV challenge (panel A) and following two HRV challenges separated by one week time (panel B). Panel C shows the peak levels in the first four days post-challenge in the group that received placebo, followed by a HRV challenge (bars 1 and 2), and in the group that were challenged with HRV twice (bars 3 and 4). Data are represented as geometric mean and 95% CI.

Viral load in nasal wash increased following HRV infection (Fig 3). At day 7, viral load was still increased, and remained at a similar level upon a second challenge with the same virus (Fig 3).

### Cytokines in nasal wash

**Fig 4:**
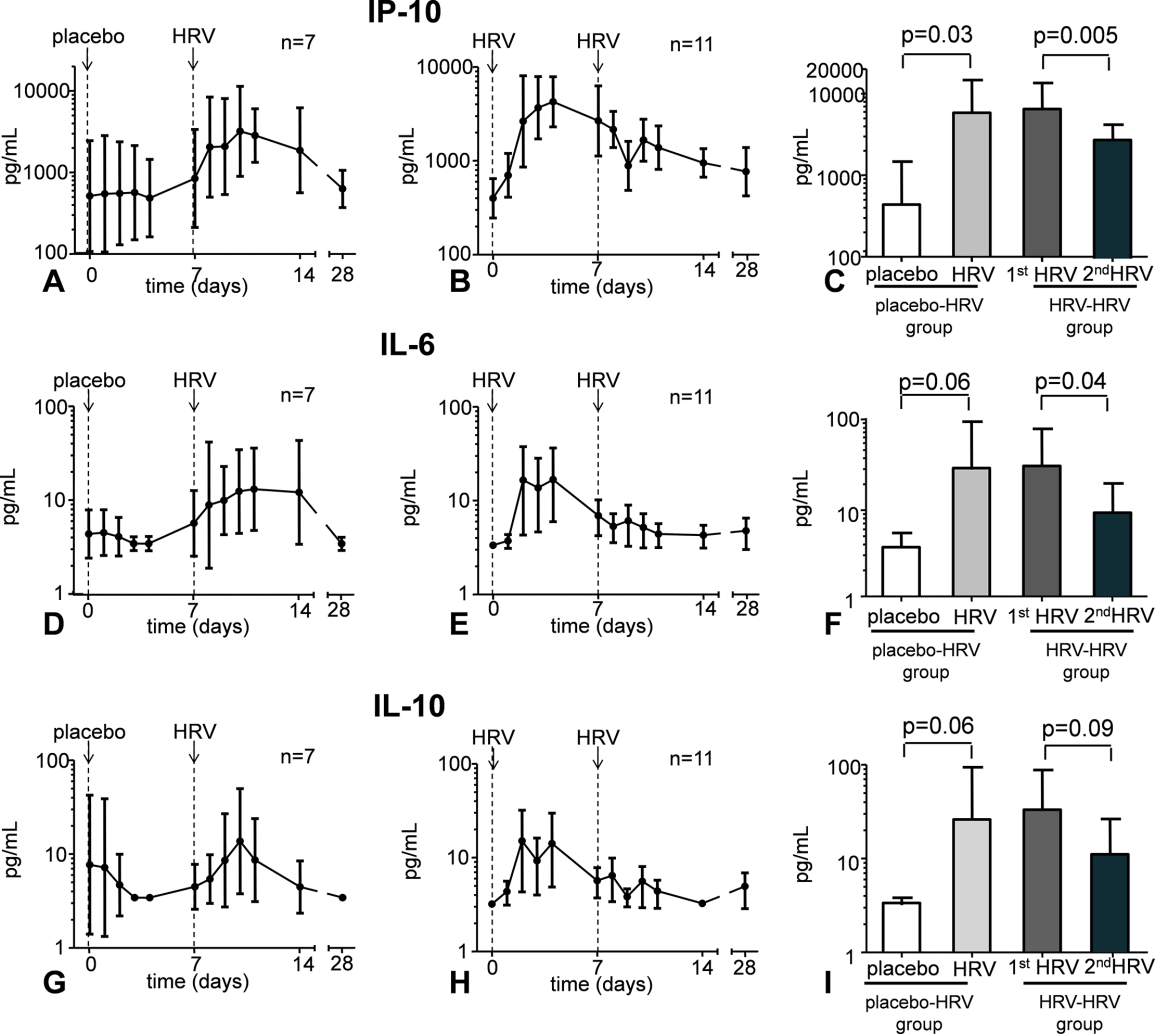
Cytokine levels in nasal wash following placebo inoculation and HRV challenge (panels A, D, and G) and following two HRV challenges separated by one week time (panels B, E, and H). Panels C, F, and I show the peak levels in the first four days post-challenge in the group that received placebo, followed by a HRV challenge (bar 1 and 2), and in the group that were challenged with HRV twice (bar 3 and 4). Data are represented as geometric mean and 95% CI. Lower detection limits were 3.2 pg/mL for IL-6 and IL-10, and 156 pg/mL for IP-10.

HRV infection did not increase the production of IFN-γ, TNF-α, IL-1β and IL-8. However, a transient increase in levels of IP-10 in nasal wash, and a similar trend for IL-6 and IL-10 was observed (Fig 4). In subjects who were challenged with HRV twice, the first HRV inoculation resulted in an identical immune response compared with subjects who received HRV once (Fig 4).

However, following the second HRV challenge, levels of IP-10 and IL-6 in nasal wash showed no further increase, but decreased significantly. A similar trend was observed for IL-10 (Fig 4). IFN-γ, IL-1β, and TNF-α levels in nasal wash were below the detection limit in virtually all subjects, and no clear profiles were observed following HRV infection.

### Systemic and lower respiratory tract responses

**Fig 5:**
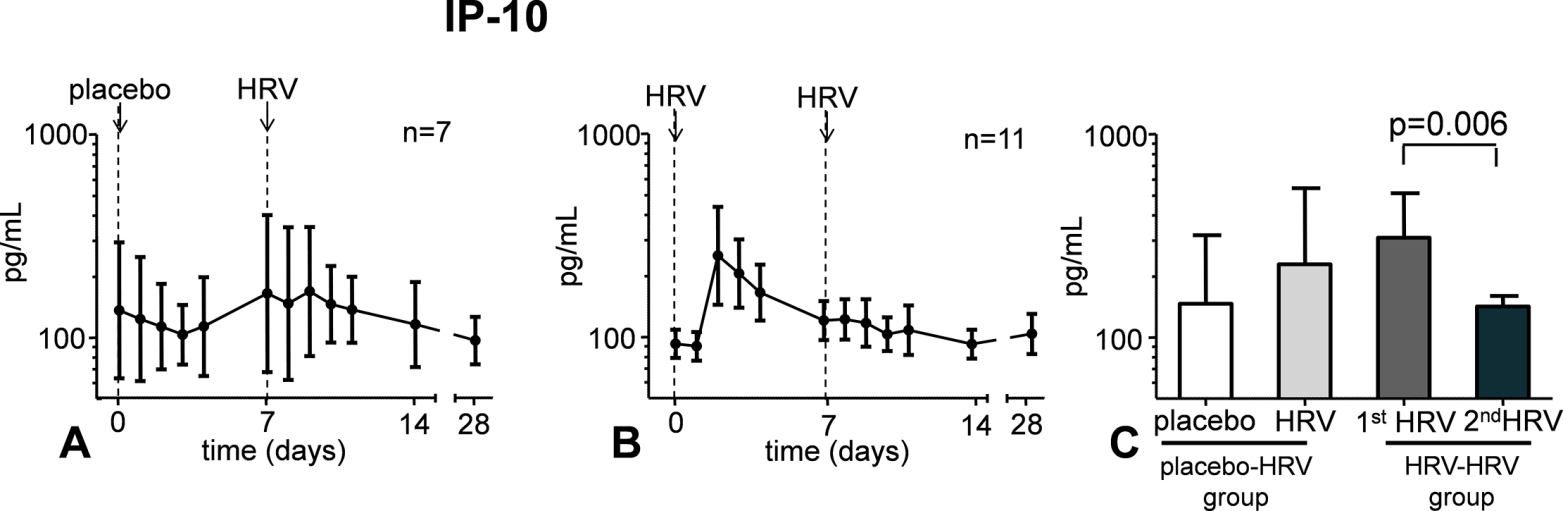
Plasma IP-10 levels following placebo inoculation and HRV challenge (panel A) and following two HRV challenges separated by one week time (panel B). Panel C shows the peak levels in the first four days post-challenge in the group that received placebo, followed by a HRV challenge (bar 1 and 2) and in the group that were challenged with HRV twice (bar 3 and 4). Data are represented as geometric mean and 95% CI. Lower detection limit was 156 pg/mL.

Plasma levels of IFN-γ, TNF-α, IL-1β, IL-6, IL-8, and IL-10 were below the detection limit in virtually all subjects at all time points. In the few subjects who displayed detectable levels of these cytokines, values were very low (approximately 10 pg/mL), in most cases already present as baseline, and did not increase following HRV infection. IP-10 levels were detectable in plasma already at baseline, but HRV infection after placebo inoculation did not result in significantly increased plasma levels (Fig 5). In subjects who were challenged with HRV twice, a more pronounced increase was observed after the first inoculation, although this response was not significantly different compared with that observed in subjects who received HRV once. Upon the second HRV challenge however, a significant decrease was observed compared with the first (Fig 5).

HRV infection resulted neither in systemic symptoms, nor in symptoms indicating a descending respiratory tract infection (Figure A in S1 File). Furthermore, HRV infection did not affect PEF, as all subjects produced values >80% of their individual predicted values at all time-points, and PEF did not change after HRV challenge (Figure B in S1 File).

## Discussion

In the present study, we show that the response of several innate immune parameters is less pronounced upon a second HRV challenge seven days after the first challenge.

We chose to investigate the influence of two consecutive HRV challenges within a short time frame. As a consequence, the observed attenuated response of several innate immune parameters to the second HRV exposure may be the result of tolerance. These results indicate that viruses causing relatively mild illness also induce attenuation of innate immune responses and may lead to increased vulnerability towards secondary fungal / bacterial infections, or reactivation of viruses that reside latent in the human host[4]. This could have consequences for vulnerable patient groups, such as the elderly, and might influence vaccination strategies. This finding is supported by *in vitro* studies that have shown that HRV can induce immunosuppressive mechanisms[22, 23]. The effects observed in the present study are reminiscent of “endotoxin tolerance”, observed after challenging healthy volunteers with bacterial lipopolysaccharide[36, 37], and might resemble some aspects of the severely immunosuppressed state observed in patients with sepsis[3] and influenza infections[5]. As such, it is tempting to speculate that previous infection with relatively mild viruses such as HRV renders patients increased vulnerable towards secondary bacterial and/or viral infections. However, additional studies are warranted to assess the clinical relevance of our observations. The tolerance effect observed could be due to desensitization effects on local immune cells or a type-I interferon induced CD8 T-cell mediated anti-viral state where no replication occurs[38]. For instance, it was shown that HRV can survive in alveolar macrophages and impair the cytokine responses to a second challenge with bacterial ligands[23]. Along these lines, for influenza and respiratory syncytial virus (RSV), previous work has demonstrated a post-viral desensitization of alveolar macrophages to Toll-like receptor (TLR) ligands, associated with reduced NF-kappaB activation and chemokine production[5].

In our study, we only included seronegative subjects, because previous work has demonstrated that serostatus alters both HRV-induced symptom scores[16, 39] as well as the HRV-induced immune response, which we showed to be virtually nullified in seropositive subjects[29]. As such, next to the pathophysiological consequences, our results indicate that a crossover design is neither feasible using a short interval, due to a suppressed innate immune response, nor using a longer interval, due to antibody formation that starts approximately one week after HRV infection[16] and severely impacts the immune response[29].

The mucosal immune response observed in the present study, reflected by the increase in cytokines in nasal wash, is in line with various previous *in vitro* and *in vivo* studies[11–13, 40]. In response to HRV infection, IP-10 was in contrast to other cytokines, produced in nasal wash of all subjects following infection. As such, our results are in accordance with previous studies that have demonstrated that IP-10 is a sensitive marker for HRV infection[12, 41, 42]. IP-10 plasma levels did not significantly increase upon a single HRV challenge although a trend was apparent, especially following the first HRV inoculation in subjects who were challenged with HRV twice. In addition, more subjects that were challenged twice demonstrated positive infection (11 in the HRV-HRV group vs. 7 in the placebo-HRV group; Fig 2). Apparently in this short time frame, the host is not yet capable to induce an effective innate immune response to eliminate HRV, supportive of the immunological tolerance theory. The mild increase of IP-10 plasma levels is in line with studies in asthmatic and COPD patients[41, 42]. In the absence of alterations of all other systemic markers measured, this relatively minor increase might suggest spillover from the nasopharynx to the circulation, although production by cells outside the nasopharynx cannot be excluded.

Apart from the increase in IP-10, neither systemic symptoms or immune effects were found after HRV infection, nor effects on the lower respiratory tract were observed. These findings suggest that, unlike what has been described for vulnerable groups such as young children, immunocompromised patients, and/or patients with pre-existing airway diseases[18–21], HRV-16 does not exert these effects in healthy subjects.

This study has several limitations. First, we included a relatively low number of subjects, which probably explains that several differences did not reach statistical significance.

Second, we only studied healthy subjects, therefore the observed effects could be different in a diseased population, such as those suffering from sepsis, which might display dysregulated cytokine responses[37].

Third, we did not store the nasal wash cellular fraction and therefore could not assess cellular markers such as induction of antiviral genes such as type I or III interferons, which play important roles in respiratory viral infections[43].

Finally, we chose to perform the second HRV challenge one week after the first, as at this time point the innate immune response induced by the first infection is expected to be largely resolved, but apparently, viral load and levels of the cytokines in nasal wash had not returned to baseline yet. However, one would expect a further increase in these parameters after a second HRV challenge in case of a normal immune response, while a similar viral load and further decrease in cytokines was apparent. In addition, although tolerance is a plausible explanation for the attenuated immune response, one could speculate about the possibility that, albeit in relatively little quantities, anti-HRV specific antibodies are already present at this time, which could also lead to attenuation of the immune response upon the second HRV-16 challenge. However, studies that investigated kinetics of HRV-induced neutralizing antibodies make this theory less likely[44, 45]. These studies demonstrate that HRV-neutralizing antibodies in both nasal wash and serum are low seven days after HRV inoculation, and their titers begin to rise at approximately two weeks after inoculation[44], although it should be mentioned that the conventional detection tests used in these studies might be less sensitive in detecting HRV-specific antibodies than the tests that are used nowadays. Nonetheless, our data also show that viral load remained elevated until day 28, when the majority of subjects displayed seroconversion. Therefore, it appears not to be possible to find a suitable time-window to investigate the effects of consecutive HRV challenges without taking into account the possibly confounding effects of the development of an adaptive immune response, leading to the production of anti-HRV specific antibodies. Using a different HRV strain for the second HRV challenge would eliminate the possible influence of HRV-16-specific antibodies on the immune response.

In conclusion, we report that a second HRV challenge one week after the first results in a less pronounced response of several innate immune parameters. This could indicate that relatively mild viruses can also induce immunosuppression, possibly leading to increased vulnerability towards secondary infections.

## Supporting information

S1 File Supporting Tables and Figures. (DOC)

S2 File Study_protocol. (DOC)

S3 File CONSORT 2010 checklist. (DOC)
