# Supplementary material for: Short-term repeated HRV-16 exposure results in an attenuated immune response in vivo in humans
Source: PLoS One. 2018 Feb 15;13(2):e0191937. doi: 10.1371/journal.pone.0191937 (PMC5813921; doi:10.1371/journal.pone.0191937)
Supplement: S1 File — (DOC) [file pone.0191937.s001.doc]

**S1 File. Supporting Tables and Figures**

**Supplementary tables**

**Table A.** Demographic characteristics of all 40 subjects. Parameters were assessed during the screening visit. M:male, F:female. BMI: body mass index. S-: seronegative for HRV-16, S+: seropositive for HRV-16. Data are presented as medians [interquartile range].

|  | **Placebo-HRV**  **M (n=10)** | **Placebo-HRV**  **F (n=10)** | **HRV-**  **HRV**  **M (n=10)** | **HRV-**  **HRV**  **F (n=10)** | **Total**  **group**  **(n=40)** | **p value between groups** |
| --- | --- | --- | --- | --- | --- | --- |
| **Age, yrs** | 23[21-25] | 24[20-26] | 22[21-25] | 23[22-24] | 22[21-25] | *1.00* |
| **Height, cm** | 187[191-190] | 168[164-173] | 185[178-190] | 173[168-176] | 178[168-186] | *0.45* (M)  *0.17* (F) |
| **Weight, kg** | 78[77-89] | 67[62-72] | 77[72-84] | 66[57-68] | 73[66-80] | *0.20* (M)  *0.62* (F) |
| **BMI, kg/m^2^** | 23[22-25] | 23[22-24] | 23[22-24] | 21[20-24] | 23[22-24] | *0.73* (M)  *0.14* (F) |
| **Serostatus** | S- (n=5)  S+ (n=5) | S- (n=6)  S+ (n=4) | S- (n=6)  S+ (n=4) | S- (n=5)  S+ (n=5) | S- (n=22)  S+ (n=18) |  |

**Table B.** Validated primer and probe-mixes for HRV used in the multiplex assay. The stem structure of the molecular beacon is italicised. Reporter dye: carboxy-fluorescein (FAM); Quencher: black hole quencher 1 (BHQ1). Probe: Taqman probe (TQ).

| Target | Primer | Sequence and label | PCR product size (bp) |
| --- | --- | --- | --- |
| HRV | 235HRVs | GACARGGTGTGAAGSYC | 142 |
|  | 236HRVas | CAAAGTAGTYGGTCCCATCC |  |
|  | 522HRV-TQ-FAM | FAM-TCCTCCGGCCCCTGAATGYGGCTAA-BHQ-1 |  |

**Table C.** HRV-16 pre-, and post-infection antibody titers of all 40 subjects. S-: seronegative for HRV-16, S+: seropositive for HRV-16.

| Group of subjects | Antibody titer  Pre-infection | Antibody titer  Post-infection | Seroconversion  yes / no |
| --- | --- | --- | --- |
| Males placebo-HRV  S- n=5  S+ n=5 | <4  <4  <4  <4  <4  8  4  4  8  45 | 4  4  388  4  32  16  91  16  8  152 | no  no  yes  no  yes  no  yes  yes  no  yes |
| Females placebo-HRV S- n=6  S+ n=4 | <4  <4  <4  4  4  4  8  128  4  8 | 64  4  4  54  23  64  8  128  8  16 | yes  no  no  yes  yes  yes  no  no  no  no |
| Males HRV-HRV S- n=6  S+ n=4 | <4  <4  <4  <4  <4  <4  64  16  4  45 | 4  8  16  16  256  64  64  19  32  128 | no  no  yes  yes  yes  yes  no  no  yes  yes |
| Females HRV-HRV S- n=5  S+ n=5 | <4  <4  <4  <4  4  8  16  16  64  27 | 4  4  1024  64  32  609  609  128  64  256 | no  no  yes  yes  yes  yes  yes  yes  no  yes |

**Supplementary figures**

**
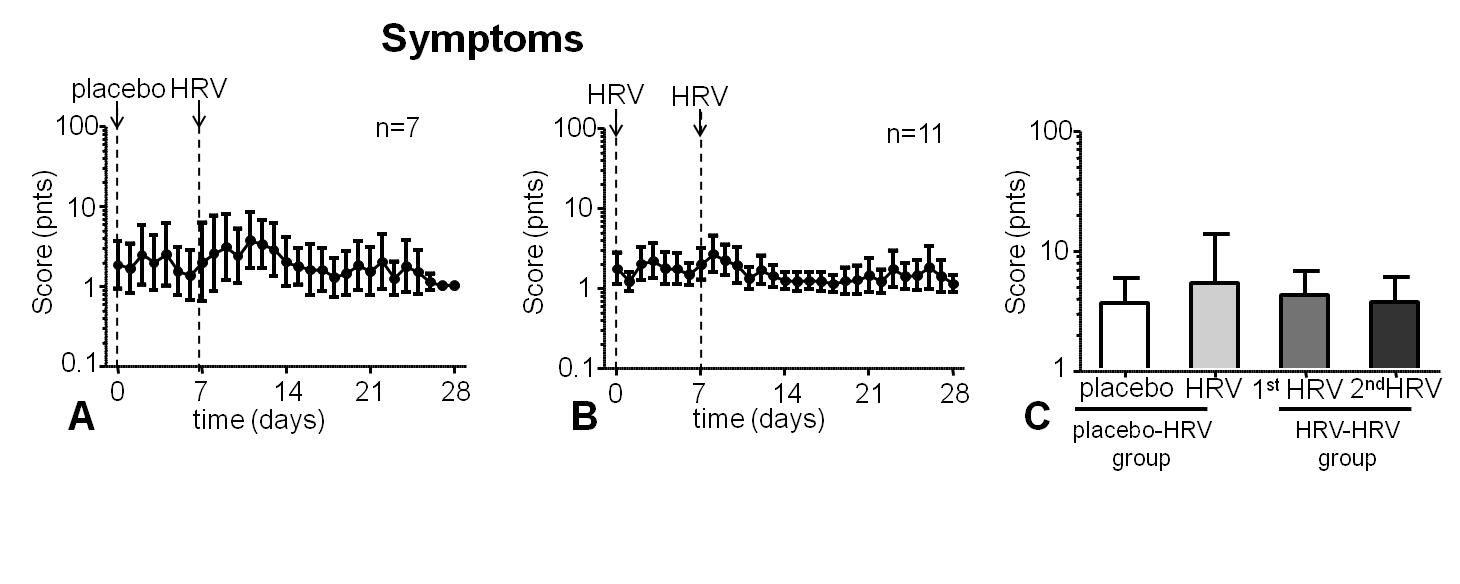
**

**Fig A.** Systemic symptom score (points) following placebo inoculation and HRV challenge (panel A) and following two HRV challenges separated by one week time (panel B). Panel C shows the peak levels in the first four days post-challenge in the group that received placebo, followed by a HRV challenge (bars 1 and 2), and in the group that were challenged with HRV twice (bars 3 and 4). Data are represented as geometric mean and 95% CI.

**
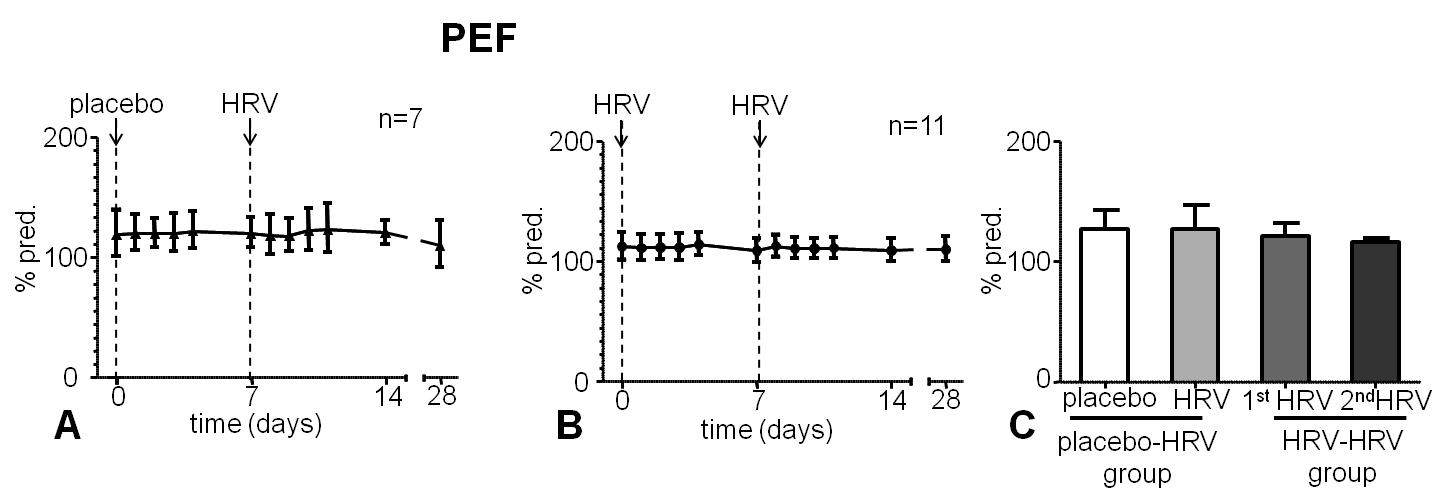
**

**Fig B.** Peak Expiratory Flow (PEF) values (% pred.) following placebo inoculation and HRV challenge (panel A) and following two HRV challenges separated by one week time (panel B). Panel C shows the peak levels in the first four days post-challenge in the group that received placebo, followed by a HRV challenge (bars 1 and 2), and in the group that were challenged with HRV twice (bars 3 and 4). Data are represented as geometric mean and 95% CI.
