# Supplementary material for: Short-term repeated HRV-16 exposure results in an attenuated immune response in vivo in humans
Source: PLoS One. 2018 Feb 15;13(2):e0191937. doi: 10.1371/journal.pone.0191937 (PMC5813921; doi:10.1371/journal.pone.0191937)
Supplement: S2 File — (DOC) [file pone.0191937.s002.doc]

**S1 FILE**

**RESEARCH PROTOCOL**

**Version 1**

***Experimental Human Rhinovirus Infection,***

***a randomized placebo-controlled pilot study***

**(October 2012)**

**PROTOCOL TITLE**

***Experimental Human Rhinovirus Infection, a randomized placebo-controlled pilot study***

| **Protocol ID:** | EHRVI |
| --- | --- |
| **Short title:** | Experimental human rhinovirus infection |
| **EudraCT number** | 2012-004938-42 |
| **Version :** | Version 1 |
| **Date :** | October 2012 |
| **Coordinating investigator/project leader:** | Prof. Dr. P. Pickkers, Internist-intensivist  Radboud University Nijmegen Medical Centre  Department of Intensive Care Medicine, PO box 9101, 6525 GA Nijmegen, The Netherlands |
| **Principal investigator:** | Drs. R. Koch, anesthesioloog i.o.  Radboud University Nijmegen Medical Centre  Department of Intensive Care Medicine, PO box 9101, 6525 GA Nijmegen, The Netherlands |
| **Associate investigators:** | Dr. M. Kox, wetenschappelijk onderzoeker  Radboud University Nijmegen Medical Centre  Department of Intensive Care Medicine, PO box 9101, 6525 GA Nijmegen, The Netherlands  Dr. Gerben Ferwerda, internist  Radboud University Nijmegen Medical Centre  Department of Pediatric infectious disease  6525 GA Nijmegen, The Netherlands  Prof. Dr. P.W.M. Hermans, Hoogleraar Kindergeneeskunde  Radboud University Nijmegen Medical Centre  Department of Pediatric Infectious Diseases, PO box 9101,  6525 GA Nijmegen, The Netherlands  Dr. Foekje Stelma, Viroloog  Radboud University Nijmegen Medical Centre  Department of Virology, PO box 9101  6525 GA Nijmegen, The Netherlands |
| **Sponsors :** | Radboud University Nijmegen Medical Centre,  Postbus 9101  6500 HB Nijmegen |
| **Subsidising party** | EFRO grant (This study is performed in the context of the Immunoforce consortium, which consist of biotechnical companies, european EFRO funding, and academic funding). |
| **Independent expert:** | Drs. A. Rennings, internist  Radboud University Nijmegen Medical Centre  Department of Internal Medicine  6525 GA Nijmegen, The Netherlands |
| **Laboratory sites:** | Department of Clinical Chemistry  Radboud University Nijmegen Medical Centre  Postbus 9101, 6500 HB Nijmegen, the Netherlands |
|  | Department of Medical Microbiology  Radboud University Nijmegen Medical Centre  Postbus 9101, 6500 HB Nijmegen, the Netherlands |
|  | Laboratory of Paediatric infectious diseases  Radboud University Nijmegen Medical Centre  Postbus 9101, 6500 HB Nijmegen, the Netherlands |
|  | Laboratory of NIZO food research BV  P.O. Box 20 6710 BA Ede, The Netherlands |

**PROTOCOL SIGNATURE SHEET**

| **Name** | **Signature** | **Date** |
| --- | --- | --- |
| *Head of Department:*  Prof. Dr. J.G. van der Hoeven,  Internist-intensivist |  |  |
| *Coordinating Investigator/Project leader:*  Prof. Dr. P. Pickkers,  Internist-intensivist |  |  |
| *Principal Investigator:*  Drs. R. Koch  Anesthesioloog i.o. |  |  |

LIST OF ABBREVIATIONS AND RELEVANT DEFINITIONS

| ABR | ABR form (General Assessment and Registration form) is the application form that is required for submission to the accredited Ethics Committee (ABR = Algemene Beoordeling en Registratie) |
| --- | --- |
| AE | Adverse Event |
| AR | Adverse Reaction |
| CA | Competent Authority |
| CCMO  CD-  COPD | Central Committee on Research Involving Human Subjects  Cluster of differentiation (a glycoprotein expressed on the surface of immune cells)  Chronic Obstructive Pulmonary Disease |
| CV | Curriculum Vitae |
| DSMB | Data Safety Monitoring Board |
| EU | European Union |
| EudraCT  GMP  HMGB1  HRV | European drug regulatory affairs Clinical Trials GCP Good Clinical Practice  Good Medical Practice  High mobility group box 1  Human Rhinovirus |
| IB | Investigators Brochure |
| IC  IFN-  Ig- | Informed Consent  Interferon-  Immunoglobulin |
| IMP | Investigational Medicinal Product |
| IMPD  IL-  LPS  MAP  MVS | Investigational Medicinal Product Dossier  Interleukin-  Lipopolysaccharide  Mean arterial pressure  Master Viral Seed |
| METC  NK  qPCR  RUNMC | Medical research ethics committee (MREC); in Dutch: medisch ethische toetsing commissie (METC)  Natural Killer  quantitative polymerase Chain Reaction  Radboud University Nijmegen Medical Centre |
| SAE | Serious Adverse Event |
| SPC | Summary of Product Characteristics (in Dutch: officiële productinformatie IB1-tekst) |
| Sponsor | The sponsor is the party that commissions the organisation or performance of the research, for example a pharmaceutical company, academic hospital, scientific organisation or investigator. A party that provides funding for a study but does not commission it is not regarded as the sponsor, but referred to as a subsidising party. |
| SUSAR  TCID_50_  VP | Suspected Unexpected Serious Adverse Reaction  Tissue Culture Infectious Dose that produces pathological change in 50% of cell cultures  Viral Protein |
| Wbp | Personal Data Protection Act (in Dutch: Wet Bescherming Persoonsgevens) |
| WMO  WURSS | Medical Research Involving Human Subjects Act (Wet Medisch-wetenschappelijk Onderzoek met Mensen  Wisconsin Upper Respiratory Symptom Survey |
|  |  |

**TABLE OF CONTENTS**

1 INTRODUCTION AND RATIONALE [10](#__RefHeading___Toc338753534)

2 OBJECTIVES [11](#__RefHeading___Toc338753535)

3 STUDY DESIGN [12](#__RefHeading___Toc338753536)

4 STUDY POPULATION [13](#__RefHeading___Toc338753537)

4.1 Population (base) [13](#__RefHeading___Toc338753538)

4.2 Inclusion criteria [13](#__RefHeading___Toc338753539)

4.3 Exclusion criteria [13](#__RefHeading___Toc338753540)

4.4 Sample size calculation [13](#__RefHeading___Toc338753541)

5 TREATMENT OF SUBJECTS [14](#__RefHeading___Toc338753542)

5.1 Investigational product / treatment [14](#__RefHeading___Toc338753543)

5.2 Use of co-intervention [15](#__RefHeading___Toc338753544)

5.3 Escape medication [15](#__RefHeading___Toc338753545)

6 INVESTIGATIONAL MEDICINAL PRODUCT [16](#__RefHeading___Toc338753546)

6.1 Name and description of investigational product(s) [16](#__RefHeading___Toc338753547)

6.2 Summary of findings from non-clinical studies [16](#__RefHeading___Toc338753548)

6.3 Summary of findings from clinical studies [16](#__RefHeading___Toc338753549)

6.4 Summary of known and potential risks and benefits [16](#__RefHeading___Toc338753550)

6.5 Description and justification of route of administration and dosage [16](#__RefHeading___Toc338753551)

6.6 Dosages, dosage modifications and method of administration [16](#__RefHeading___Toc338753552)

6.7 Preparation and labeling of Investigational Medicinal Product [17](#__RefHeading___Toc338753553)

6.8 Drug accountability [17](#__RefHeading___Toc338753554)

7 METHODS [18](#__RefHeading___Toc338753555)

7.1 Study parameters/endpoints [18](#__RefHeading___Toc338753556)

7.1.1 Main study parameter/endpoint: [18](#__RefHeading___Toc338753557)

7.1.2 Secondary study parameters/endpoints: [18](#__RefHeading___Toc338753558)

7.1.3 Other study parameters [18](#__RefHeading___Toc338753559)

7.2 Randomization, blinding and treatment allocation [19](#__RefHeading___Toc338753560)

7.3 Study procedures (Flowchart) [19](#__RefHeading___Toc338753561)

7.4 Withdrawal of individual subjects [22](#__RefHeading___Toc338753562)

7.4.1 Specific criteria for withdrawal [22](#__RefHeading___Toc338753563)

7.5 Replacement of individual subjects after withdrawal [22](#__RefHeading___Toc338753564)

7.6 Follow-up of subjects withdrawn from treatment [22](#__RefHeading___Toc338753565)

7.7 Premature termination of the study [23](#__RefHeading___Toc338753566)

8 SAFETY REPORTING [24](#__RefHeading___Toc338753567)

8.1 **Section 10 WMO event** [24](#__RefHeading___Toc338753568)

8.2 **Adverse and serious adverse events** [24](#__RefHeading___Toc338753569)

*8.2.1* *Adverse event* [24](#__RefHeading___Toc338753570)

*8.2.2* *Serious Adverse Events* [24](#__RefHeading___Toc338753571)

*8.2.3* *Suspected unexpected serious adverse reactions (SUSAR)* [24](#__RefHeading___Toc338753572)

8.3 **Annual safety report** [25](#__RefHeading___Toc338753573)

8.4 **Follow-up of adverse events** [26](#__RefHeading___Toc338753574)

8.5 **Data Safety Monitoring Board (DSMB)** [26](#__RefHeading___Toc338753575)

8.6 **Abnormal laboratory tests results** [26](#__RefHeading___Toc338753576)

9 STATISTICAL ANALYSIS [28](#__RefHeading___Toc338753577)

9.1 Modified intention to treat analysis [28](#__RefHeading___Toc338753578)

9.2 Descriptive statistics [28](#__RefHeading___Toc338753579)

9.3 Multivariate analysis [28](#__RefHeading___Toc338753580)

9.4 Interim analysis [28](#__RefHeading___Toc338753581)

10 ETHICAL CONSIDERATIONS [29](#__RefHeading___Toc338753582)

10.1 Regulation statement [29](#__RefHeading___Toc338753583)

10.2 Recruitment and consent [29](#__RefHeading___Toc338753584)

10.3 Compensation for injury [29](#__RefHeading___Toc338753585)

10.4 Benefits and risks assessment, group relatedness [29](#__RefHeading___Toc338753586)

10.5 Incentives [30](#__RefHeading___Toc338753587)

11. ADMINISTRATIVE ASPECTS, MONITORING AND PUBLICATION [31](#__RefHeading___Toc338753588)

12. STRUCTURED RISK ANALYSIS [33](#__RefHeading___Toc338753589)

12.1 Potential issues of concern [33](#__RefHeading___Toc338753590)

12.2 Synthesis [33](#__RefHeading___Toc338753591)

13. REFERENCES [34](#__RefHeading___Toc338753592)

**SUMMARY**

**Title** Experimental Human Rhinovirus Infection, a randomized placebo-controlled pilot study

**Rationale** The importance of the common cold derives primarily from its frequency and from its enormous socioeconomic impact. Human Rhinoviruses (HRVs) are the major cause of the common cold, being responsible for 30-50% of all acute respiratory illnesses with no causal remedies at hand. A model to investigate the pathophysiology of HRV infection and to test compounds that could treat or protect one from infection or developing symptoms would therefore be very valuable. With this HRV model it is also possible to investigate crosstalk between bacteria and viruses. This is very relevant because, following a viral infection, bacterial superinfections are common in clinical practice, and underlying mechanisms and subsequent possible therapies that could prevent this remain to be discovered. Worldwide, thousands of subjects have been exposed to experimental rhinovirus infection, of which more than 600 to HRV-16. Serious adverse events related to rhinovirus infection have never been documented. Therefore, this model can be considered a safe and highly reproducible model. Moreover, 52 volunteers have already been exposed to the HRV-16 virus from the batch that we want to use in this study.

**Objective** Our primary objective is to set up the Human Rhinovirus (HRV)-model in our centre. In addition, to facilitate future clinical trials, we want to determine optimal read-out parameters and read-out time points for experimental HRV infection in healthy volunteers. Furthermore, we want to analyze to what extent HRV can cause systemic immune effects, and we want to test if subjects with antibodies against HRV can be re-infected with the same virus, and if the presence of HRV antibodies influences the local clinical and immunological response upon infection. Additionally, to gain insight in the immune modulating properties of HRV, we want to investigate the immunological response to a HRV re-infection within one week to determine if there are mechanisms that provide immediate protection against re-infection. This facilitates a cross-over design of future pharmacological intervention-trials. Furthermore, we want to investigate the capacity of HRV infection to modulate the systemic immune response by analyzing the response of leukocytes *ex vivo* stimulated with different stimuli. Moreover, we want to evaluate the effects of HRV-16 infection on the host transcriptome and metabolome. Finally, The influence of HRV-16 infection on nasal and gut microbiota will be assessed.

**Study design** A parallel randomized double-blind placebo-controlled pilot study.

**Study population** Healthy volunteers (n=40), 18-35 years of age.

**Intervention** Healthy volunteers who meet all inclusion criteria and none of the exclusion criteria that have given informed consent to participate in the study will be randomized to become either inoculated with HRV-16 (n=20; 10 male+10 female) or with placebo (lactated Ringer’s solution, n=20, 10 male+ 10 female). After one week a second inoculation with HRV-16 will be performed in both groups. In both groups, 50% of the subjects will be sero-negative and 50% sero-positive to HRV-16. One hundred TCID_50_ units of HRV-16 (by spraying 0.5 mL into each nostril in supine position in a randomized manner) will be administered.

**Main parameters /endpoints** The main study parameter is the rate of infection (defined by a positive viral culture, qPCR and/or a four-fold rise in antibody titre) caused by HRV-16 inoculation. Secondary endpoints include the duration of the incubation period, the effects of HRV-16 (re-)infection on cold symptoms and spirometry, kinetics of HRV-16-induced local inflammation parameters in nasal washes (including immune cells and cytokine production), kinetics of the HRV-16-induced systemic immune response (including circulating cytokines), the ability of HRV to modulate the systemic immune response (as reflected by the *ex vivo* production of inflammatory mediators by stimulated leukocytes), the effects of seropositivity on clinical and immunological responses, and the effects of HRV-16 infection on faecal and nasal-pharyngeal microbiota and host transcriptome and metabolome.

**Nature and extent of the burden and risks associated with participation, benefit and group relatedness:**

Subjects have to visit the hospital on a total of 13 occasions (about 10 minutes each visit). Upon screening, a medical interview is conducted. Subjects have to keep a symptoms diary (scoring card) that they fill in at home. In total, maximally 500 ml of blood will be drawn (on 13 occasions via venapuncture). This is not associated with side effects (500 mL is also drawn at the blood bank without any side effects). Furthermore, 12 nasal washes will be performed, which can lead to slight irritation of the nasal mucosa but is not associated with risks, and subjects have to collect faecal samples on four occasions. HRV infection is associated with short-term symptoms of a cold. Worldwide, thousands of subjects have been exposed to experimental rhinovirus infection, of which more than 600 to HRV-16. Serious adverse events related to rhinovirus infection have never been documented. Therefore, this model can be considered a safe and highly reproducible model. Moreover, 52 volunteers have already been exposed to the HRV-16 virus from the same batch that we intend to use in this study.

# INTRODUCTION AND RATIONALE

The common cold is a benign and self-limiting illness . The importance of the common cold derives primarily from its high frequency and from its enormous socioeconomic impact . Human Rhinoviruses (HRVs) are the major cause of the common cold, being responsible for 30-50% of all acute respiratory illnesses . HRV infections occur worldwide, causing symptoms such as sneezing, coughing, sore throat, nasal / head congestion and tiredness. To date, it is unknown what mechanisms provide protection against a second HRV-infection in the short term, before development of antibodies, and whether the presence of antibodies against HRV actually prevent re-infection with the same virus or modulate the immune response to the virus. Moreover, it is unknown to what extend HRV infection causes systemic immunological effects, and to what extent HRV can modulate the immune response (locally or remotely) to a subsequent infection with bacteria or other viruses. Investigating this crosstalk is highly relevant because following a viral infection, bacterial superinfections are common in clinical practice (for example a staphylococcus aureus pneumonia after an influenza infection and the underlying mechanisms and subsequent possible therapies that could prevent this remain to be investigated. Furthermore, the role of the composition of the gut microbiota, which has recently been shown to play a pivotal mediating role in virus infections in susceptibility to HRV infection is unknown.

To investigate the common cold in a controlled manner, an experimental HRV infection model was developed in the early seventies of the previous century . In this model, healthy volunteers are infected with a standardized virus dose . Over the past four decades, many trials have used this model to investigate the effects of HRV infection on symptoms , pathogenesis , local immunity , pulmonary function , allergies , asthma and COPD exacerbations, and to measure the effects of preventive and therapeutic interventions (see ATTACHMENT 1). In the present pilot study, we wish to set up the Human Rhinovirus model to investigate the hitherto unknown effects of HRV infection mentioned in the first paragraph. In addition, the HRV infection model could also be used to investigate possible anti-viral therapies, or the effects of nutritional supplements on HRV infection.

*Study proposal:*

We want to perform a double-blind placebo-controlled pilot study with the primary objective to set up the Human Rhinovirus (HRV)-model in healthy volunteers in our centre, using HRV serotype 16 (HRV-16). In addition, to facilitate future clinical trials, we want to determine optimal read-out parameters and read-out time points for experimental HRV infection. Furthermore we want to analyze to what extent HRV causes systemic effects, if subjects with antibodies against HRV can be re-infected with the same virus, and whether the presence of HRV antibodies influences the clinical and immunological response upon infection. To gain insight in the immune modulating properties of HRV, we want to examine the immunological response to a HRV re-infection within one week to determine if there are mechanisms (and which) that provide short-term protection against re-infection. In addition, we want to investigate the capacity of HRV infection to modulate immune responses by circulating leukocytes by analyzing the response of leukocytes *ex vivo* stimulated with different TLR-agonists. Finally, the gut microbiota will be analyzed to investigate its relation with the immune response and susceptibility towards HRV infection and vice versa (effects of HRV infection on microbiota).

# OBJECTIVES

*Primary objective***:** To set up the Human Rhinovirus (HRV)-model in our centre, using HRV serotype 16 (HRV-16). The primary outcome measure is the infection rate (defined by a positive viral culture, qPCR and/or a four-fold rise in antibody titre) of healthy volunteers inoculated with a standardized dose of HRV-16.

*Secondary Objectives:* there are 10 secondary objectives**:**

1. To determine the incubation period of HRV-16 infection.
2. To determine the effects of HRV-16 infection on cold symptoms, temperature, and spirometry.
3. To determine suitable inflammatory parameters (and their kinetics) that play a role in the HRV-16-induced local inflammatory response by measuring leukocyte counts and differentiation as well as cytokine levels in nasal washes.
4. To determine whether HRV-16 infection can induce a systemic immune response and its kinetics by measuring leukocyte counts and differentiation, and circulating plasma cytokines.
5. To determine the effect of HRV-16 seropositivity on the local + systemic clinical and immunological response to re-infection with HRV-16.
6. To determine if a subject is protected against re-infection with HRV-16 within one week (tolerance formation to HRV), and if so which mechanisms play a role (local immune response parameters, systemic immune response parameters).
7. To determine whether HRV-16 infection modulates the immune response of circulating leukocytes by measuring the cytokine response, of leukocytes *ex vivo* stimulated with different inflammatory stimuli.
8. To determine the effects of HRV-16 (re-)infection on the nasal, oral and faecal microbiota.
9. To determine the effects of HRV-16 (re)infection on the host transcriptome.
10. To determine the effects of HRV-16 (re)infection on the host metabolome.

# STUDY DESIGN

A parallel, randomized placebo-controlled pilot study in healthy male and female volunteers. The subjects will be randomized to become either inoculated with HRV-16 (n=20; 10 male + 10 female) or with placebo (lactated Ringer’s solution n=20, 10 male + 10 female). After one week a second inoculation with HRV-16 will be performed in both groups. Study procedures are provided in detail in section 7.4.

# STUDY POPULATION

## Population (base)

The study population will consist of 40 healthy, non-smoking male and female volunteers (1:1), age 18-35 years, 50% sero-negative and 50% sero-positive to HRV-16 at screening. Before inclusion, subjects must meet all inclusion criteria and none of the exclusion criteria. All volunteers must have given informed consent to participate in the study. Recruitment of healthy volunteers will take place by placement of posters in the medical faculty and several other faculties and locations on the campus of the Radboud University Nijmegen.

## Inclusion criteria

- Age ≥18 and ≤35 years of age
- Healthy
- Use of contraceptives (for female subjects only)

## Exclusion criteria

- Pregnancy or lactating
- Pre-existent lung disease, including asthma
- A history of allergic rhinitis
- Use of any medication
- Use of alcohol > 5/day or >20/wk
- Use of any drugs
- Current smoker or more than 5 pack-year history
- Frequently have nosebleeds
- Recent nasal or otologic surgery
- Febrile illness or a common cold within four weeks before the HRV challenge
- Currently participating in another clinical trial
- Use of antibiotics, norit, laxatives (up till 6 months prior to inclusion), cholestyramine, acid burn inhibitors or immune suppressive agents (up till 3 months prior to inclusion), and pre- and probiotics (up till 1 month prior to inclusion).

## Sample size calculation

The present study is a pilot study to set up the Human Rhinovirus model. No interventions are tested against each other or against a placebo. Therefore, a power calculation is not warranted.

# TREATMENT OF SUBJECTS

## Investigational product / treatment

Human Rhinovirus-16 (HRV-16)

Although HRV is not an Investigational Medicinal Product (IMP), we felt that a proper description of the preparation that has been used so far is required to substantiate its safety and efficacy in clinical studies and, therefore, we have used the format of the IB from the CCMO website as an outline for this section. Furthermore, we have also submitted the IB for HRV-16.

*General Properties:* HRVs are non-enveloped, single-stranded RNA viruses that belong to the Picornaviridae family . They have linear, single stranded, positive-sense RNA genomes of between 7.2 and 8.5kb in length. The viral particles themselves are not enveloped and are icosahedral in structure with a diameter of approximately 30nm. The capsid of HRVs is composed of 60 copies of each of the four capsid proteins: VP1, VP2, VP3 and VP4 . Three of these proteins are located on the surface of the capsid and are responsible for the antigenic diversity of HRV, whereas the fourth, VP4, is much smaller and located inside the virion . Of the four viral capsid proteins, VP1 is the most exposed and immunodominant surface protein. It is critically involved in the infection of respiratory cells and is predominantly recognized by HRV neutralizing antibodies Currently, >100 distinct serotypes are primarily assigned to two genetic species, HRV-A and HRV-B, with >70% amino acid identity and a similar antiviral susceptibility pattern within each species . Recently, a third species, HRV-C, including a number of previously unrecognized non-cultivable HRVs, has been identified In addition, the serotypes have also been classified into major and minor groups according to the receptor they use to enter epithelial cells of the respiratory tract. Members of the major receptor group bind to the intercellular adhesion molecule (ICAM)-1 and the representatives of the minor receptor group use members of the low-density lipoprotein receptor family .

The two HRV subtypes most used in the Human Rhinovirus infection model are HRV-39 and HRV-16 (ATTACHMENT 1). In this study we will use HRV-16 to inoculate healthy volunteers because this subtype is currently the only virus available that is produced according to good manufacturing practice (GMP) (see IB). Up to date, over 30 human trials (with over 600 HRV infected subjects) have been published with HRV-16 (ATTACHMENT 1). Exposure to HRV-16 by inoculation induces cold symptoms in 80-90% of those exposed. The time point for onset of symptoms but not the severity of symptoms is dependent on the viral load (expressed as TCID_50_; dose of virus that produces pathological change in 50% of cell cultures) used for inoculation . It is unknown whether HRV-16 is endemic in this region, and, if so, how many people have antibodies against HRV-16.

*Provider:*

##### Respivert Ltd

##### Imperial BioIncubator

##### level 1 Bessemer Building (RSM)

##### Prince Consort Road

##### London

##### SW7 2BP, United Kingdom

## Use of co-intervention

The use of any medication during this study is considered an exclusion criterion.

## Escape medication

Subjects that will receive HRV will be under supervision of an experienced medical practitioner. No escape medications are applicable in this study.

# INVESTIGATIONAL MEDICINAL PRODUCT

Although HRV is not an Investigational Medicinal Product (IMP), we felt that a proper description of the preparation that has been used so far is required to substantiate its safety and efficacy in clinical studies and, therefore, we also submitted the IB of the HRV-16 batch.

## Name and description of investigational product(s)

Human Rhinovirus 16 (HRV-16). HRV-16 will be diluted in a total volume of 0.5 mL 0.9% saline and inoculated via the nasal route. Subjects will receive 100 TCID_50_ units of HRV-16 at study day 1 and / or day 7.

## Summary of findings from non-clinical studies

Not applicable. HRV-16 has been administered to hundreds of healthy volunteers worldwide.

## Summary of findings from clinical studies

Summary of findings from clinical studies are listed in the IB. HRV-16 has been administered to hundreds of healthy volunteers worldwide, is considered safe and no longterm effects have ever been documented.

## Summary of known and potential risks and benefits

Summary of potential risks and side effects are listed in the IB. Up to date over 30 human trials (with over 600 inoculated subjects) have been published with HRV-16 (ATTACHMENT 1). No serious adverse effects have ever been documented (ATTACHMENT 1). Therefore, this intervention is considered safe. HRV induces short term cold symptoms and exposure is not associated with any long term side-effects. Subjects will not benefit directly from participation to the study. A subject fee is provided.

## Description and justification of route of administration and dosage

Intranasal administration is a regular, non-invasive route of administration (see IB). Route of administration and dosages are based on previous studies.

## Dosages, dosage modifications and method of administration

See 6.5.

## Preparation and labeling of Investigational Medicinal Product

A stock of Human Rhinovirus A, type 16 (HRV-16) will be provided by Respivert according to GMP standards in 200 μL aliquots of 40 TCID_50_ per aliquot. Five aliquots of 200 μL will be pooled to obtain 200 TCID_50_ of HRV-16 per mL. This stock will be pretitered (1:1) with ringers lactate and the obtained 2 mL will be separated in two tubes of 1 mL and stored at -80^o^C at the virology department of the Radboud University Medical Centre. After freezing, one tube will be thawed, cultured and titered in order to estimate the TCID_50_ after storage at -80°C.

At each day of infection, one tube will be thawed for each subject and divided in two aliquots of 0.5 mL, one aliquot for each nostril. Further, per infection day one aliquot of 0.5 mL HRV-16 will be cultured in order to control infectivity, and one aliquot will be submitted to semi-quantitative RT-PCR analysis in order to have a molecular t_0_ reference value.

The aliquots with HRV-16 or placebo will be labelled by an independent research nurse of the intensive care research unit.

## Drug accountability

A qualified person (QP) of the department of virology of the Radboud University Nijmegen Medical Centre will sign off the drug accountability form. This form will list which HRV vial is used for which subject(s) on what date. Used vials will be stored at the virology department until completion of the trial (including data analysis).

# METHODS

## Study parameters/endpoints

### Main study parameter/endpoint:

The primary outcome measure is the infection rate (defined by a positive viral culture, qPCR and/or a four-fold rise in antibody titre) of healthy volunteers inoculated with a standardized dose of HRV-16.

### Secondary study parameters/endpoints:

- Incubation period of HRV-16 infection.
- Symptom scores measured by diary cards (WURSS 21 scoring method).
- Temperature.
- Forced expiratory volume at a timed interval of 1 second (FEV1), and forced expiratory flow 25-75% (FEF 25-75%).
- Leukocyte counts and differentiation (NK-cells, CD4 / CD8, neutrophils), and cytokine levels in nasal washes (including but not limited to IL-8, IL-1β, CCL5).
- Leukocyte counts and circulating plasma cytokines (including but not limited to TNF-α, IL-6, IL-10, IFN-γ, IL-8, CCL5).
- The cytokine response (including but not limited to TNF-α, IL-6, IL-10, IFN-γ), of leukocytes *ex vivo* stimulated with different stimuli (including but not limited to LPS, HRV, Staphylococcus aureus).
- Composition of the gut microbiota.
- The host transcriptome and metabolome.
- Composition of the nasal-pharyngeal microbiota.

### Other study parameters

Demographic characteristics (age, body weight, height) will be recorded.

## Randomization, blinding and treatment allocation

Healthy volunteers who meet all inclusion criteria and none of the exclusion criteria that have given informed consent to participate in the study will be randomized to become either inoculated with HRV-16 (n=20; 10 male + 10 female) or with placebo (lactated Ringer’s solution, n=20, 10 male + 10 female). Subjects will be stratified according to age and serotype. After one week, a second inoculation with HRV-16 will be performed in both groups. At the beginning of the study, subjects will be allocated to the placebo+HRV or the HRV+HRV group by the opening of sealed envelopes by a research nurse not involved in the study. HRV-16 or placebo will be delivered in identical packs prepared by an independent nurse. Code of the randomization is kept by the independent research nurse and will be broken only if necessary for safety reasons. The investigators and participating subjects will be blinded until the study endpoints have been determined. Only subjects belonging to one group (placebo or HRV) will be inoculated together. This to prevent infection of the placebo-group by unintended exposure through contact with HRV-infected subjects. The first group of 10 subjects will be inoculated, two weeks later (when the first group of subjects is not infectious any more) a second group will be inoculated, and so on.

## Study procedures (Flowchart)

For flowcharts see APPENDIX 1 and 2. The day of virus challenge will be denoted as day 0. Once entered, the subject remains in the study for 28 days. The period from viral challenge until week 4, is defined as the “study period”. All subjects will be assessed according to clinical and immunological criteria at regular intervals during and at the end of the study period. A baseline assessment will be conducted prior to randomization into the study and viral challenge. Blood sampling and nasal washes will be collected at different time points during the follow-up.

*Screening visit, within one month before HRV inoculation:*

Before inclusion in the study, volunteers will be subjected to a medical interview during the screening visit. During this visit, all study procedures will be explained to the subjects, informed consent will be obtained and a medical history (including prior engagement in studies, and allergic and other adverse reactions during hospital admissions) will be documented. Blood will withdrawn to determine serum neutralizing antibody to specific challenge virus strains. Subjects are eligible for the study if they meet all inclusion and none of the exclusion criteria. A container for faecal sampling will be given to the volunteers.

*Daily throughout the study period (day 0-28):*

all subjects will keep a diary (WURSS 21) in which they record cold symptoms, fever, and other possible complaints, twice daily for the first two weeks and once daily in the last 2 weeks of the study.

*Randomization and inoculation (day 0):*

At day 0, subjects will visit the Intensive Care research department. Subjects have to blow their nose and body temperature will be measured by an ear-thermometer. Subjects will be inoculated in a standardized fashion (10^2^ TCID_50_ units of HRV-16 diluted in a total volume of 0.5 mL 0.9% saline, or 0.5 mL lactated Ringer’s solution [placebo] will be administered into each nostril using a pipette). The subjects will be in supine position and are instructed to inhale deeply through the nose. This procedure will be repeated a number of times until all of the inoculum is instilled). TCID_50_ will be measured parallel to inoculation by viral culture. Before virus inoculation, blood will be obtained by vena puncture and a nasal wash is performed. Previous research has shown that concerning the nasal sampling methods (wash, aspirate, swab and brush), the nasal wash procedure has the highest detection rate for HRV, without any relevant discomfort for the subject . For the nasal wash procedure a syringe filled with 5 mL of prewarmed 0.9% sodium chloride will be inserted into one of the nasal cavities and the volunteer will be instructed to take a deep breath and hold it for 10 seconds. Next, the volunteer will lean forward and blows the fluid gently through both nares onto a sterile Petri dish. The procedure will be repeated with the other naris and the contents will be pooled into a universal container. To enhance virus stability a concentrated virus transport buffer will be added. Furthermore, all volunteers will bring a faecal sample in a container produced for this purpose, provided by NIZO and frozen at -80°C until analyzed at NIZO. All subjects will start recording their symptoms in diaries. After 10 minutes, all measurements will be finished and subjects leave the hospital wearing facemasks to guard in-hospital patient safety (see chapter safety).

*First acute cold phase (days 1,2, 3, and 4):*

All subjects will visit the Intensive Care research department. Subjects have to blow their nose and body temperature will be measured. Blood will be withdrawn by venapuncture and nasal washes will be performed. After 10 minutes, all measurements will be finished and subjects leave the hospital. At day 2, all volunteers will bring their faecal sample.

*Days 5, 6, 12 and 13*

At these days, volunteers will record their symptoms twice a day.

*Convalescence and second inoculation (day 7):*

At day 7, subjects will be admitted to the Intensive Care research department. Subjects have to blow their nose and body temperature will be measured. All subjects will receive HRV-16 by nasal spray in each nostril in supine position as described above. TCID_50_ will be measured parallel to inoculation by viral culture. Before virus inoculation, blood will be obtained by venapuncture and a nasal wash is performed. Furthermore, all volunteers will bring their faecal sample. After 10 minutes, all measurements will be finished and subjects leave the hospital.

*Second acute cold phase ( day 8, day 9, day 10, and day 11):*

All subjects will be admitted to the Intensive Care research department. Subjects have to blow their nose and body temperature will be measured. Blood will be drawn and nasal washes will be performed. After 10 minutes, all measurements will be finished and subjects leave the hospital.

*Convalescence (day 14, and day 28):*

All subjects will be admitted to the Intensive Care research department. Subjects have to blow their nose and body temperature will be measured. Blood will be drawn and nasal washes will be performed. After 10 minutes, all measurements will be finished and subjects leave the hospital. Recording of symptom in diaries will end at day 28. Seroconversion will be measured at day 28.

*Methods of determination*

Upon inoculation, a portion of the used inoculums will be used for quantitative virus culture, to ensure that the inoculum is indeed infective (see section 6.7). This will be performed at the virology department of the Radboud University Nijmegen Medical Centre.

To determine the severity of the cold symptoms, all subjects fill out a cold symptom diary based on the WURSS 21 scoring method which is a standardized and frequently used scoring diary card in experimental HRV studies (ATTACHMENT 2). During the acute cold phases, they record symptoms in their diaries twice per day (in the morning and in the evening), followed by once per day after day 14 until day 28. Diary cards will be scored for sore throat, sneezing, rhinorrhea, nasal congestion, wheeze, dyspnoea, chest tightness, and cough. Symptoms will be scored on a scale ranging from 0, (symptom not present) to 7 (worst ever experienced). At days 7 and 14 subjects will be asked whether they experienced a cold or not in the previous days.

Nasal washes will be performed after symptom scoring, by 5 mL prewarmed 0.9% sodium chloride into one of the nasal spaces. The volunteer briefly holds their breath while the 5 mL is expelled into the nasal space. After 10 seconds, the volunteer leans forward and blows the fluid gently through the nares into a sterile petri dish. Next, the nasal wash fluid will be transferred to a test tube to assess volume and will be vortexed and centrifuged, creating a cell-free supernatant, stored at –80°C for later analysis.

The incubation period will be measured by the time between HRV inoculation and start of cold symptoms.

Viral load will be determined by RNA isolation, followed by qPCR and/or virus culture at the virology department of the Radboud University Nijmegen Medical Centre.

Seroconversion will be determined by the presence of neutralizing antibody to the challenge virus, measured in serum at the department of virology of the Radboud University Nijmegen Medical Centre. Seroconversion to HRV-16 antibody is determined by comparison sera taken on day 28 with the baseline value. Subjects with a fourfold increase in antibody titer will be considered infected.

Phenotyping of immune cells (NK-cells, CD4 / CD8, neutrophils) will be determined by flowcytometry at the Laboratory of Paediatric Infectious Diseases of the Radboud University Nijmegen Medical Centre.

The measurements of the various cytokines (including TNF-α, IL-6, IL-10, IL-8, IL-1β, IFN-γ, and CCL5) , in serum and/or nasal washes will be determined by Luminex multiplex assays and ELISA according to manufacturer’s instructions at the Laboratory of Paediatric Infectious Diseases and the department of Intensive Care Medicine of the Radboud University Nijmegen Medical Centre.

*Ex vivo* leukocyte stimulation will be performed at the Laboratory of Paediatric Infectious Diseases of the Radboud University Nijmegen Medical Centre. Cytokines (TNF-α, IL-6, IL-10, and IFN-γ) in supernatants of stimulated leukocyte cultures will be determined by ELISA according to manufacturer’s instructions. Stimuli used include, but are not limited to LPS, HRV, and staphylococcus aureus.

Microbiota analysis of the gut will be determined by micro-array and will be performed at NIZO. At T=-1/0, 2/3, 6/7, 9/10 and 13/14, faecal samples will be collected. All materials and information needed for proper collection of the faecal samples (stool collection kit) will be supplied by NIZO food research and delivered to the subjects. Faeces will be frozen immediately after defaecation. Subjects will be asked to store faeces in mini-freezers, supplied by NIZO food research. Every 2-3 days, the frozen faeces will be transported to the lab, weighed, and homogenized. Homogenized faecal sub-samples will be frozen and stored (at -20 ˚C) for later analyses.

Microbiota analysis of the nasal-pharyngeal wash will be determined by micro-array and will be performed at the Laboratory of Paediatric Infectious Diseases.

The host transcriptome and metabolome analysis will be performed at NIZO.

## Withdrawal of individual subjects

Subjects can withdraw from the study at any time without any consequences and without the need to give an explanation if they wish to do so. The investigator can decide to withdraw a subject from the study for medical reasons. Subject that withdraw during the study will receive a proportional fee.

### Specific criteria for withdrawal

When the subject has given informed consent, the subject is irrevocably admitted to the trial. Even if the subject is withdrawn from the study, documentation according to the study protocol must be as complete as possible.

A subject can or will be withdrawn from the study:

- upon request of the subject
- after protocol violation
- at the discretion of the investigator

## Replacement of individual subjects after withdrawal

After withdrawal or exclusion of a subject, he or she will be replaced to maintain adequate power of the study.

## Follow-up of subjects withdrawn from treatment

After withdrawal, subjects are monitored if medically necessary for the time of the study protocol. For all subjects who are prematurely withdrawn from treatment, the reason will be documented carefully and the general practitioner will be informed. For all subjects who were withdrawn after randomization, but before treatment with study medication, the reason why will be documented.

## Premature termination of the study

The coordinating investigators have the right to discontinue the clinical study at any time for medical or procedural reasons.

# SAFETY REPORTING

## **Section 10 WMO event**

In accordance to section 10, subsection 1, of the WMO, the investigator will inform the subjects and the reviewing accredited METC if anything occurs, on the basis of which it appears that the disadvantages of participation may be significantly greater than was foreseen in the research proposal. The study will be suspended pending further review by the accredited METC, except insofar as suspension would jeopardise the subjects’ health. The investigator will take care that all subjects are kept informed.

## **Adverse and serious adverse events**

### *Adverse event*

Adverse events are defined as any undesirable experience occurring to a subject during a clinical trial, whether or not considered related to the investigational drug. All adverse events reported spontaneously by the subject or observed by the investigator or his staff will be recorded.

### *Serious Adverse Events*

A serious adverse event is any untoward medical occurrence or effect that at any dose:

- results in death;
- is life threatening (at the time of the event);
- requires hospitalisation or prolongation of existing inpatients’ hospitalisation;
- results in persistent or significant disability or incapacity;
- is a congenital anomaly or birth defect;
- Any other important medical event that may not result in death, be life threatening, or require hospitalization, may be considered a serious adverse experience when, based upon appropriate medical judgement, the event may jeopardize the subject or may require an intervention to prevent one of the outcomes listed above.

The principal investigator will report the SAEs through the web portal *ToetsingOnline* to the accredited METC that approved the protocol, within 15 days after the principal investigator has first knowledge of the serious adverse reactions.

SAEs that result in death or are life threatening should be reported expedited. The expedited reporting will occur not later than 7 days after the principal investigator has first knowledge of the adverse reaction. This is for a preliminary report with another 8 days for completion of the report.

### *Suspected unexpected serious adverse reactions (SUSAR)*

Adverse reactions are all untoward and unintended responses to an investigational product related to any dose administered.

This investigation does formally not involve an investigational medicinal product, because HRV is designated as a non-investigational medicinal product. Nevertheless, analogous to our previous human endotoxemia trials, we choose to include the SUSAR paragraph (HRV-related) in the present protocol.

Unexpected adverse reactions are SUSARs if the following three conditions are met:

1. the event must be serious;
2. there must be a certain degree of probability that the event is a harmful and an undesirable reaction to the medicinal product under investigation, regardless of the administered dose;
3. the adverse reaction must be unexpected, that is to say, the nature and severity of the adverse reaction are not in agreement with the product information as recorded in:

- Summary of Product Characteristics (SPC) for an authorised medicinal product;
- Investigator’s Brochure for an unauthorised medicinal product.

The principal investigator will report expedited the following SUSARs through the web portal *ToetsingOnline* to the METC:

- SUSARs that have arisen in the clinical trial that was assessed by the METC;
- SUSARs that have arisen in other clinical trials of the same sponsor and with the same medicinal product, and that could have consequences for the safety of the subjects involved in the clinical trial that was assessed by the METC.

The remaining SUSARs are recorded in an overview list (line-listing) that will be submitted once every half year to the METC. This line-listing provides an overview of all SUSARs from the study medicine, accompanied by a brief report highlighting the main points of concern. The expedited reporting of SUSARs through the web portal ToetsingOnline is sufficient as notification to the competent authority.

The principal investigator will report expedited all SUSARs to the competent authorities in other Member States, according to the requirements of the Member States.

The expedited reporting will occur not later than 15 days after the principal investigator has first knowledge of the adverse reactions. For fatal or life threatening cases the term will be maximal 7 days for a preliminary report with another 8 days for completion of the report.

## **Annual safety report**

In addition to the expedited reporting of SUSARs, the principal investigator will submit, once a year throughout the clinical trial, a safety report to the accredited METC, competent authority, and competent authorities of the concerned Member States.

This safety report consists of:

- a list of all suspected (unexpected or expected) serious adverse reactions, along with an aggregated summary table of all reported serious adverse reactions, ordered by organ system, per study;
- a report concerning the safety of the subjects, consisting of a complete safety analysis and an evaluation of the balance between the efficacy and the harmfulness of the medicine under investigation.

## **Follow-up of adverse events**

All adverse events will be followed until they have abated, or until a stable situation has been reached. Depending on the event, follow up may require additional tests or medical procedures as indicated, and/or referral to the general physician or a medical specialist.

## **Data Safety Monitoring Board (DSMB)**

A DSMB is established for this study to perform ongoing safety surveillance and to perform interim analyses on the safety data. the DSMB is an independent committee completely unblinded for treatment allocation and is composed of the following persons:

Prof. dr. G.J. Scheffer, anaesthesist

Radboud University Nijmegen Medical Centre

Department of Anesthesia, PO box 9101, 6500 HB Nijmegen, The Netherlands

Prof. Dr. M. Netea, internist, infectioloog

Radboud University Nijmegen Medical Centre

Department of Medicine, Nijmegen Institute for Infection, Inflammation and Immunity (N4i), PO box 9101, 6500 HB Nijmegen, The Netherlands

The first interim analysis will be performed after 10 subjects have completed the study, a second interim analysis after 20 subjects have completed the study and the last interim analysis will be performed after 30 subjects have completed the study. The DSMB members will assess safety in a unblinded manner.

A DSMB-charter is attached (ATTACHMENT 3). The interim analysis will focus on the following issues: adverse events: type, severity, duration, action taken and attributability to experimental HRV-infection.

The following criteria are defined on which basis the DSMB may decide to terminate the trial prematurely:

- Any serious adverse Event

## **Abnormal laboratory tests results**

The results of all laboratory tests required by the protocol will be recorded in the subjects’ case record form. All clinically important abnormal laboratory tests occurring during the study will be repeated at appropriate intervals until they return either to baseline or to a level deemed acceptable by the investigator, or until a diagnosis that explains them is made.

# STATISTICAL ANALYSIS

## Modified intention to treat analysis

Not applicable.

## Descriptive statistics

For the descriptive statistics, values will be given as mean ± SD or median and ranges, depending on their distribution. For comparisons, paired Students t -tests or Willcoxon-tests for parametric and nonparametric data as appropriate will be used. Changes over time will be analysed using ANOVA repeated measures or friedman tests as appropriate. A p-value <0.05 is considered significant. Statistical analyses will be performed with SPSS 16.0 (SPSS, Chicago, IL) software.

## Multivariate analysis

Not applicable.

## Interim analysis

Not applicable.

# ETHICAL CONSIDERATIONS

## Regulation statement

The study will be conducted according to the principles of the Declaration of Helsinki and in accordance with the Medical Research Involving Human Subjects Act (WMO).

## Recruitment and consent

Recruitment of healthy volunteers will take place by placement of posters in the medical faculty and several other faculties and locations on the campus of the Radboud University Nijmegen. Candidate subjects will receive written information. Thereafter an informative meeting will be organized for each subject at which they will be informed about the study set-up, and information will be given on the dietary restrictions, the symptoms diaries, the rhinovirus infection, and the collection of faeces and blood. Subjects will be given at least 1 day to consider their decision. If positive, written informed consent will be obtained and after that subjects will be scheduled for screening to determine their eligibility based on the in- and exclusion criteria of the study.

## Compensation for injury

The sponsor/investigator has a liability insurance which is in accordance with article 7, subsection 6 of the WMO.

The sponsor has an insurance which is in accordance with the legal requirements in the Netherlands (Article 7 WMO and the Measure regarding Compulsory Insurance for Clinical Research in Humans of 23th June 2003). This insurance provides cover for damage to research subjects through injury or death caused by the study.

1. € 450.000,-- (i.e. four hundred and fifty thousand Euro) for death or injury for each subject who participates in the Research;
2. € 3.500.000,-- (i.e. three million five hundred thousand Euro) for death or injury for all subjects who participate in the Research;
3. € 5.000.000,-- (i.e. five million Euro) for the total damage incurred by the organisation for all damage disclosed by scientific research for the Sponsor as ‘verrichter’ in the meaning of said Act in each year of insurance coverage.

The insurance applies to the damage that becomes apparent during the study or within 4 years after the end of the study.

## Benefits and risks assessment, group relatedness

The subjects will not benefit directly from participation to the study. A subject fee of 400 euro is provided.

Subjects have to visit the hospital on a total of 13 occasions (about 10 minutes each visit). Upon screening, a medical interview is conducted. Subjects have to keep a symptoms diary. In total, maximally 500 ml of blood will be drawn (on 13 occasions via venapuncture). This drawn amount of blood is not associated with side effects (500 mL is also drawn at the blood bank without any side effects). Furthermore, 12 nasal washes will be performed, which can lead to slight irritation of the nasal mucosa but is not associated with risks. HRV infection is associated with mild short-term symptoms of a cold. Worldwide, thousands of subjects have been exposed to experimental rhinovirus infection, of which more than 600 to HRV-16. Serious adverse events related to rhinovirus infection have never been documented. Therefore, this model can be considered a safe and highly reproducible model. Moreover, 52 volunteers have already been exposed to the HRV-16 virus from the batch that we intent to use in this study.

Because of the potential value of the experimental HRV infection model, with the possibility to investigate the pathophysiology of HRV, crosstalk between viral and bacterial infections, and to test compounds that could treat or prevent the major cause of the common cold worldwide, being responsible for 30-50% of all acute respiratory illnesses with currently no causal remedies for hand, we feel that the risk to, and burden for the subjects are in proportion to the potential value of the research.

A structured risk assessment is provided in section 12.

## Incentives

Subjects will be compensated with 400 Euro after completion of the study. If the subject ends participation before completion of the study the subject will receive a proportional share of the total fee. When the subject is forced to stop because of medical reason before the end of the study, the complete amount will be paid.

# 11. ADMINISTRATIVE ASPECTS, MONITORING AND PUBLICATION

11.1 Handling and storage of data and documents

Data will be handled confidentially and anonymously. A subject identification code list will be used to link the data to the subject. The code is not based on the patient initials and birth-date. The code used will be HRV1.XX, where XX is the number of the subject based on the order of the screening visit (first screened subject is HRV1.01 etc.).

The key to the code will be safeguarded by the principal investigator.

The handling of subject data in this study complies with the Dutch Personal Data Protection Act (in Dutch: De Wet Bescherming Persoonsgegevens, WBP).

11.2 Monitoring and Quality Assurance

Monitoring will be carried out by an internal monitor of the Radboud University Nijmegen Medical Centre after completion of the study. The stored data in the CRFs, all informed consents, SAE reports and the trial master file will be monitored.

11.3 Amendments

Not applicable.

11.4 Annual progress report

The principal investigator will submit a summary of the progress of the trial to the accredited METC once a year. Information will be provided on the date of inclusion of the first subject, numbers of subjects included and numbers of subjects that have completed the trial, serious adverse events/ serious adverse reactions, other problems, and amendments.

11.5 End of study report

The principal investigator will notify the accredited METC and the competent authority of the end of the study within a period of 90 days. The end of the study is defined as the last patient’s last visit.

In case the study is ended prematurely, the sponsor will notify the accredited METC and the competent authority within 15 days, including the reasons for the premature termination.

Within one year after the end of the study, the principal investigator will submit a final study report with the results of the study, including any publications/abstracts of the study, to the accredited METC and the Competent Authority.

11.6 Public disclosure and publication policy

The principal investigator and the coordinating investigator are first and last author of the manuscript. This trial is not sponsored by a pharmaceutical company. Authors have no conflict of interest to declare. The study will be published regardless the results of the trial.

12. STRUCTURED RISK ANALYSIS

## 12.1 Potential issues of concern

*HRV inoculation*

HRV infection is accompanied by short-term mild cold symptoms. In total, thousands of subjects worldwide have been inoculated with HRV, more than 600 with HRV-16, and 52 subjects with HRV-16 derived from the batch we want to use in the present proposed study. Side effects or long-term effects have never been documented. Therefore, the HRV infection model is considered safe .

*Blood withdrawal*

In total, a maximum of 500 ml of blood will be drawn by vena puncture, which is not expected to result in side effects (same as blood donation at the blood bank).

*Nasal washes*

Nasal irrigation may cause slight irritation of the nasal passages, which is of short duration and not considered harmful. In previous studies with a similar protocol, irritation was not mentioned.

12.2 Synthesis

In our opinion, the remaining risks for participation in this study are negligible, and we have made every effort to minimize potential risks or counteract potential adverse reactions. Therefore, we feel that the remaining risks are acceptable and do not outweigh the scientific and medical relevance of this study.

13. REFERENCES

1. Harris, J.M., 2nd and J.M. Gwaltney, Jr., *Incubation periods of experimental rhinovirus infection and illness.* Clin Infect Dis, 1996. **23**(6): p. 1287-90.

2. Denny, F.W., Jr., *The clinical impact of human respiratory virus infections.* Am J Respir Crit Care Med, 1995. **152**(4 Pt 2): p. S4-12.

3. Bakaletz, L.O., *Viral potentiation of bacterial superinfection of the respiratory tract.* Trends Microbiol, 1995. **3**(3): p. 110-4.

4. Peterson, K.M., et al., *Effects of dietary supplementation with conjugated linoleic acid on experimental human rhinovirus infection and illness.* Antivir Ther, 2009. **14**(1): p. 33-43.

5. Gritzfeld, J.F., et al., *Comparison between nasopharyngeal swab and nasal wash, using culture and PCR, in the detection of potential respiratory pathogens.* BMC Res Notes, 2011. **4**: p. 122.

6. Spyridaki, I.S., et al., *Comparison of four nasal sampling methods for the detection of viral pathogens by RT-PCR-A GA(2)LEN project.* J Virol Methods, 2009. **156**(1-2): p. 102-6.

7. Mufson, M.A., et al., *Effect of Neutralizing Antibody on Experimental Rhinovirus Infection.* JAMA, 1963. **186**: p. 578-84.

8. Weidner, T.G., et al., *Effect of a rhinovirus-caused upper respiratory illness on pulmonary function test and exercise responses.* Med Sci Sports Exerc, 1997. **29**(5): p. 604-9.

9. Bardin, P.G., et al., *Peak expiratory flow changes during experimental rhinovirus infection.* Eur Respir J, 2000. **16**(5): p. 980-5.

10. Halperin, S.A., et al., *Pathogenesis of lower respiratory tract symptoms in experimental rhinovirus infection.* Am Rev Respir Dis, 1983. **128**(5): p. 806-10.

11. Igarashi, Y., et al., *Analysis of nasal secretions during experimental rhinovirus upper respiratory infections.* J Allergy Clin Immunol, 1993. **92**(5): p. 722-31.

12. Leigh, R., et al., *Human rhinovirus infection enhances airway epithelial cell production of growth factors involved in airway remodeling.* J Allergy Clin Immunol, 2008. **121**(5): p. 1238-1245 e4.

13. Wang, J.H., et al., *Infection rate and virus-induced cytokine secretion in experimental rhinovirus infection in mucosal organ culture: comparison between specimens from patients with chronic rhinosinusitis with nasal polyps and those from normal subjects.* Arch Otolaryngol Head Neck Surg, 2008. **134**(4): p. 424-7.

14. Fraenkel, D.J., et al., *Lower airways inflammation during rhinovirus colds in normal and in asthmatic subjects.* Am J Respir Crit Care Med, 1995. **151**(3 Pt 1): p. 879-86.

15. Avila, P.C., et al., *Effects of allergic inflammation of the nasal mucosa on the severity of rhinovirus 16 cold.* J Allergy Clin Immunol, 2000. **105**(5): p. 923-32.

16. Turner, R.B., et al., *Association between interleukin-8 concentration in nasal secretions and severity of symptoms of experimental rhinovirus colds.* Clin Infect Dis, 1998. **26**(4): p. 840-6.

17. Alper, C.M., et al., *Prechallenge antibodies moderate disease expression in adults experimentally exposed to rhinovirus strain hanks.* Clin Infect Dis, 1998. **27**(1): p. 119-28.

18. Bardin, P.G., et al., *Amplified rhinovirus colds in atopic subjects.* Clin Exp Allergy, 1994. **24**(5): p. 457-64.

19. Doyle, W.J., et al., *Rhinovirus 39 infection in allergic and nonallergic subjects.* J Allergy Clin Immunol, 1992. **89**(5): p. 968-78.

20. Skoner, D.P., et al., *Lower airway responses to rhinovirus 39 in healthy allergic and nonallergic subjects.* Eur Respir J, 1996. **9**(7): p. 1402-6.

21. Zambrano, J.C., et al., *Experimental rhinovirus challenges in adults with mild asthma: response to infection in relation to IgE.* J Allergy Clin Immunol, 2003. **111**(5): p. 1008-16.

22. Grunberg, K., et al., *Rhinovirus-induced airway inflammation in asthma: effect of treatment with inhaled corticosteroids before and during experimental infection.* Am J Respir Crit Care Med, 2001. **164**(10 Pt 1): p. 1816-22.

23. Halperin, S.A., et al., *Exacerbations of asthma in adults during experimental rhinovirus infection.* Am Rev Respir Dis, 1985. **132**(5): p. 976-80.

24. Kloepfer, K.M., et al., *Effects of montelukast on patients with asthma after experimental inoculation with human rhinovirus 16.* Ann Allergy Asthma Immunol, 2011. **106**(3): p. 252-7.

25. Mallia, P., et al., *Experimental rhinovirus infection as a human model of chronic obstructive pulmonary disease exacerbation.* Am J Respir Crit Care Med, 2011. **183**(6): p. 734-42.

26. Mallia, P., et al., *An experimental model of rhinovirus induced chronic obstructive pulmonary disease exacerbations: a pilot study.* Respir Res, 2006. **7**: p. 116.

27. Schoop, R., et al., *Echinacea in the prevention of induced rhinovirus colds: a meta-analysis.* Clin Ther, 2006. **28**(2): p. 174-83.

28. Cohen, S., et al., *Positive emotional style predicts resistance to illness after experimental exposure to rhinovirus or influenza a virus.* Psychosom Med, 2006. **68**(6): p. 809-15.

29. Barrett, B., et al., *The Wisconsin Upper Respiratory Symptom Survey is responsive, reliable, and valid.* J Clin Epidemiol, 2005. **58**(6): p. 609-17.

30. Gern, J.E., et al., *Rhinovirus inhibits antigen-specific T cell proliferation through an intercellular adhesion molecule-1-dependent mechanism.* J Infect Dis, 1996. **174**(6): p. 1143-50.

31. Lysholm, F., et al., *Characterization of the viral microbiome in patients with severe lower respiratory tract infections, using metagenomic sequencing.* PLoS One, 2012. **7**(2): p. e30875.

32. Wang, J.H., et al., *Staphylococcal enterotoxins A and B enhance rhinovirus replication in A549 cells.* Am J Rhinol, 2007. **21**(6): p. 670-4.

33. Spier, R.E., *On the need for, and the delivery of, cross-protective vaccines.* Vaccine, 2005. **23**(17-18): p. 2027-9.

34. Che, Z., et al., *Antibody-mediated neutralization of human rhinovirus 14 explored by means of cryoelectron microscopy and X-ray crystallography of virus-Fab complexes.* J Virol, 1998. **72**(6): p. 4610-22.

35. Barrett, B., et al., *The Wisconsin Upper Respiratory Symptom Survey (WURSS): a new research instrument for assessing the common cold.* J Fam Pract, 2002. **51**(3): p. 265.

36. Wang, J.H., H. Kim, and Y.J. Jang, *Cigarette smoke extract enhances rhinovirus-induced toll-like receptor 3 expression and interleukin-8 secretion in A549 cells.* Am J Rhinol Allergy, 2009. **23**(6): p. e5-9.

37. Ishizuka, S., et al., *Effects of rhinovirus infection on the adherence of Streptococcus pneumoniae to cultured human airway epithelial cells.* J Infect Dis, 2003. **188**(12): p. 1928-39.

38. van Eijk, L.T., M.J. Dorresteijn, and P. Pikkers, *Gender influences in vivo human responses to endotoxin.* Shock, 2008. **29**(3): p. 422; author reply 422-3.

39. Kistler, A.L., et al., *Genome-wide diversity and selective pressure in the human rhinovirus.* Virol J, 2007. **4**: p. 40.

40. Genesig, *Quantification of Human Rhinovirus 16 - genomes*, in *Polyprotein gene Standard kit handbook*2004. p. 12.

APPENDIX 1

**Flowchart 1: study procedure**

| **Time** | **Intervention** |
| --- | --- |
| Max. 2 weeks before HRV inoculation | **Screening visit**   - Medical interview - Informed consent - Blood sampling |
| Day 0 | **Prior to HRV inoculation**   - Randomization in HRV-HRV / placebo-HRV group - Nasal washes - Blood sampling - Symptom diaries - Faecal sampling within 24hrs prior to inoculation |
|  | **HRV inoculation**   - HRV inoculation |
| Day 1 | **Acute cold**   - Nasal washes - Blood sampling - 2x Symptom diaries |
| Day 2 | **Acute cold**   - Nasal washes - Blood sampling - Faecal sampling - 2x Symptom diaries |
| Day 3 | **Acute cold**   - Nasal washes - Blood sampling - 2x Symptom diaries |
| Day 4 | **Acute cold**   - Nasal washes - Blood sampling - 2x Symptom diaries |
| Day 7 | **Prior to HRV inoculation**   - Nasal washes - Blood sampling - Symptom diaries - Faecal sampling within 24hrs prior to 2^nd^ inoculation |
|  | **HRV inoculation**   - 2^nd^ HRV inoculation |
| Day 8 | **2^nd^ Acute cold**   - Nasal washes - Blood sampling - 2x Symptom diaries |
| Day 9 | **2^nd^ Acute cold**   - Nasal washes - Blood sampling - 2x Symptom diaries - Faecal sampling |
| Day 10 | **2^nd^ Acute cold**   - Nasal washes - Blood sampling - 2x Symptom diaries |
| Day 11 | **2^nd^ Acute cold**   - Nasal washes - Blood sampling - 2x Symptom diaries |
| Day 14 | **Convalescence**   - Nasal washes - Blood sampling - Symptom diaries - Faecal sampling |
| Day 28 | **Convalescence**   - Nasal washes - Blood sampling - Symptom diaries |

APPENDIX 2

***Flowchart 2: measurements***

| Time point | Screening  (D≤-14) | D0 | D1 | D2 | D3 | D4 | D5 | D6 | D7 | D8 | D9 | D10 | D11 | D12 | D13 | D14 | D28 |
| --- | --- | --- | --- | --- | --- | --- | --- | --- | --- | --- | --- | --- | --- | --- | --- | --- | --- |
| Medical history | X |  |  |  |  |  |  |  |  |  |  |  |  |  |  |  |  |
| Serum antibody titer | X |  |  |  |  |  |  |  |  |  |  |  |  |  |  |  | X |
| Cold symptoms (WURSS),  twice daily |  | X | X | X | X | X | X | X | X | X | X | X | X | X | X |  |  |
| Cold symptoms (WURSS),  ones daily |  |  |  |  |  |  |  |  |  |  |  |  |  |  |  | X | X |
| Temperature  Other complaints |  | X | X | X | X | X |  |  | X | X | X | X | X |  |  | X | X |
| Local response (nasal washes *) |  | X | X | X | X | X |  |  | X | X | X | X | X |  |  | X | X |
| Systemic response  (blood **) |  | X | X | X | X | X |  |  | X | X | X | X | X |  |  | X | X |
| Gut microbiota |  | X |  | X |  |  |  |  | X |  | X |  |  |  |  | X |  |

(*)viral load , cytokines , leukocyte counts, nasal-pharyngeal microbiota

(**) leukocyte counts, circulating cytokines (TNF-α, IL-6, IL-10), cytokine production by *ex vivo* stimulated leukocytes, transcriptome, and metabolome.

ATTACHMENT 1: List of studies with the experimental Human Rhinovirus infection

ATTACHMENT 2: Wisconsin Upper Respiratory Symptom Survey 21: Scoringskaart

ATTACHMENT 3: DSMB charter

ATTACHMENT 4: appointment schedule
